# Evolutionary dynamics of hyperbolic language

**DOI:** 10.1371/journal.pcbi.1010872

**Published:** 2023-02-23

**Authors:** Madison S. Krieger

**Affiliations:** Organismic and Evolutionary Biology, Harvard University, Cambridge, Massachusetts, United States of America; Dartmouth College, UNITED STATES

## Abstract

Models of evolution of simple languages have typically assumed full alignment of the speaker and listeners interests, with perfect understanding representing the optimal outcome for both parties. In more realistic settings, communicating individuals will often desire different outcomes from one another. Previous work has shown that misalignment of speaker-listener interests reduces the maximum informativeness among Nash-equilibrium languages, and that multiple equilibrium languages (with different degrees of informativeness) are supported. We study the stochastic evolutionary dynamics of signaling games in which the alignment of speaker-listener interests can vary. We find that increased misalignment of speaker-listener interests is associated with a decrease in information transmission. Moreover, the most common languages to evolve are typically the most informative languages supportable as static Nash equilibria, suggesting a solution to the ‘equilibrium selection problem’. In addition, our dynamics reveal the mechanism by which less informative languages evolve: words that previously signaled intense states come to be used hyperbolically for less intense states, with listeners’ interpretation of these newly-ambiguous words evolving downward in response. We ground our results in linguistic data on intensifiers such as *so* and *very*, words which have unique dynamics—with constant recycling and innovation that match our theoretical results well.

## Author summary

Analyses of signaling and language have typically assumed that both speakers and listeners have a common interest, such as to communicate the location of predators or resources or to convey precise instructions on how to perform a particular task. However, Nature is also rife with situations where mutual interest cannot be assumed. Many such situations have gradated states, signals, and actions, where speaker and listener desire different actions in conjunction with different true states, which are only known to the speaker. For instance, baby birds regularly overstate their true hunger level to their mother, due to a drive to obtain more than the base requirements in attention and nutrients; there are countless parallels in the context of human communication. Previous works in static game theory have established interesting results in modeling misaligned talk, such as the presence of multiple equilibria and the rapid shrinkage of lexica as misalignment of interest increases. Here we expand both the static theory, but also show using evolutionary game theory which equilibria are selected and why. We make a strong connection to linguistics via intensifiers—words such as “so”, “really”, and “very”—which suggests this literature may be immediately salient to sociolinguistics and other disciplines.

## Introduction

The evolution of simple lexical languages, in which communication is achieved by associating objects with signals, is often studied in the context of language (or “signaling”) games [1–10]. The simplest language game involves a speaker, a listener, *n* objects, and *n* words. The speaker sees an object, and speaks a word to the listener (who has not seen the object). The listener then predicts what the object is, based on the word she heard. If she correctly identifies the object, both speaker and listener receive a payoff of one point. If she does not, both get zero points. In this way, the speaker and listener’s interests are perfectly aligned [1]. From the point of view of both players, an optimal language is one-to-one: the speaker uses a different word for each object, and the listener correctly translates each word back to its unique object. In such games, evolutionary dynamics often lead to an optimal language [3–9], especially in the presence of mutations and random drift [4, 5, 7, 9].

In reality, however, the interests of communicating individuals are often imperfectly aligned. This is perhaps clearest for strategic communication in humans, where individuals often have only partially aligned preferences over the outcome of an event that their communication could influence (e.g., job applications [11, 12]). Examples of imperfect alignment of speaker-listener interests in nature are also common. For instance, bird chicks are known to vary the intensity of their begging chirps in relation to their nutritional needs [13]. Natural selection would favour mothers who feed their chicks exactly in relation to nutritional need, but it would also favour chicks who can attract slightly more than this maternal optimum, a classic example of parent-offspring conflict [14]. Similar situations are predicted to arise in foetal-maternal hormonal signalling [15] and between symbionts [16, 17]. It is therefore important to understand the effect that misalignment of interests can have on language evolution.

In a seminal paper, Crawford and Sobel studied the effect of misalignment of speaker-listener interests on the sets of Nash equilibria of static (played once-off) signalling games [2]. They found that the maximum informativeness (number of words used) among equilibrium languages decreases with the degree of speaker-listener misalignment, and that multiple languages—of varying degrees of informativeness, measured in the number of signals used—can simultaneously be supported as Nash equilibria (including the perfectly uninformative ‘babbling’ equilibrium where only one word is spoken). This latter finding raises the ‘equilibrium selection’ question: for a given degree of speaker-listener misalignment, which equilibrium language is most likely? Crawford and Sobel invoked Schelling’s concept of ‘focal points’ to argue that, for a given degree of misalignment, the most informative possible language in the equilibrium set is most ‘prominent’, and therefore most likely to be used. This argument faces serious problems in the context of signalling games: it is heuristic, does not apply to natural signalling systems, and does not identify the mechanisms by which more informative languages might come to be used [18].

Evolutionary game theory offers a potential solution to such problems. In evolutionary game theory, the dynamics of Darwinian selection and/or cognitive learning are studied, and the possible outcomes of these dynamics characterized [19, 20]. Deterministic evolutionary dynamics, the most commonly employed kind, have been used to characterize the sets of evolutionary equilibria of signalling games [3, 9], as well as to reveal how simple languages can evolve from states of non-communication [3, 6, 9]. A downside of deterministic dynamics is that, when multiple evolutionary equilibria exist, there are limited (and often only unnatural) ways to rank these equilibria—e.g., by comparing the volumes of each equilibrium’s basin of attraction in the full state space [21].

More recently, stochastic evolutionary dynamics have been developed for games [20]. These dynamics involve randomness from mutation and sampling effects, and often constitute a recurrent Markov process which visits every possible state infinitely often. The relative time that the dynamics spend in each state constitutes a natural and unambiguous way to rank states, and thus to predict which equilibria of a game are most likely [22–25]. Moreover, stochastic evolutionary dynamics allow for observation of the mechanisms by which equilibria are reached, and, in particular, the mechanisms underlying evolutionary switches between equilibria [26, 27]. This latter property is especially useful when the equilibria of a game are only neutrally stable, so that frequent switches among them are expected [26].

Here, we study the stochastic evolutionary dynamics of language games with imperfect alignment of speaker-listener interests. We aim to: (i) determine whether equilibrium languages tend to evolve in the dynamics, (ii) characterize the degree of information transmission in the most common equilibrium languages, (iii) study the associated signal dynamics when imperfectly informative equilibrium languages evolve from more informative languages and from less informative (possibly also equilibrium) languages, and (iv) explore some real-world patterns of language use to see if they are consistent with our results.

We find a prominent role for hyperbolic language in association with points (iii) and (iv) above. In keeping with the literature on linguistic pragmatics, we will distinguish between *honest* and *dishonest* hyperbole. In a crowd-sourced taxonomy of figurative language [28], the top-ranked use for hyperbole chosen by hundreds of English speakers in a variety of contexts was 1) “to clarify”. This highlights the importance of non-factual axes in human communication, since hyperbole is then the act of telling a lie in order to “clarify” the truth (honest hyperbole). For instance, consider the case of the sentence “It took a million years for us to get a table at that restaurant” [29]. The speaker is not trying to persuade the listener of the veracity of this statement; rather, an inaccurate signal (“a million years”) has been used to convey something besides the basic facts of the situation, which we might call *affect*. Affect in language has been implemented both in a game-theoretic framework [30–34] and probabilistic frameworks based on rational speech [29, 35]. However, there is a different type of hyperbolic language, revealed in the second-top-ranked use: 2) “to emphasize”. Here, that which is being emphasized need not represent the ground truth, and it is this dishonest hyperbole that will center anchor the empirical axis of our study. In particular, we focus on *intensifiers*—words like *very, so, really, pretty, amazingly, …*, which are purely emphatic, amplifying, or “boosting” in nature [36, 37]. Words like these, which are meant to increase the immediate response (“I am so very hungry” from a child to a parent) or perceived veracity (“We are amazingly talented scholars”) from the listener [38], rely on the listener’s interpretation of them as conveying an accurate amplification. However, the listener only has a finite capability to oblige this desire on the part of the speaker, leading to a situation in which the speaker desires a greater response than the listener is capable of, or cares to, provide—in other words, this type of language can explicitly correspond to a misalignment of interests between speaker and listener.

## Model

### Signaling game

An intuitive way to incorporate misalignment of interests into language games is to explicitly model how the listener acts in response to hearing each word. If, in some cases, the action that is optimal for her is different to that which is optimal for the speaker, then their interests are misaligned, with the degree of misalignment calibrated by the disparity between their respective optimal outcomes. This requires a notion of ‘distance’ between outcomes, and so it is natural to assume that the words and actions in the game are quantitative, with the listener wanting more than the speaker wants to give.

Formally, then, suppose that the state (or ‘object’) observed by the speaker, *s*, comes from the set $S=\{s_{1},s_{2},\ldots ,s_{I}\}\subset R$, with *s*_1_ < *s*_2_ < … < *s*_*I*_. These states could describe an intensity of hunger, for instance, or how qualified a candidate is for a job. State *s*_*i*_ occurs with probability *p*_*i*_. The speaker, having observed the state, speaks a word *w* to the listener, with the word chosen from the set $W=\{w_{1},w_{2},\ldots ,w_{J}\}\subset Z$. The listener, having heard the word spoken, chooses an action *a* from the set $A=\{a_{1},a_{2},\ldots ,a_{K}\}\subset R$, with *a*_1_ < *a*_2_ < … < *a*_*K*_. These actions might represent an amount of food given, or a salary offered, etc.

**Fig 1:**
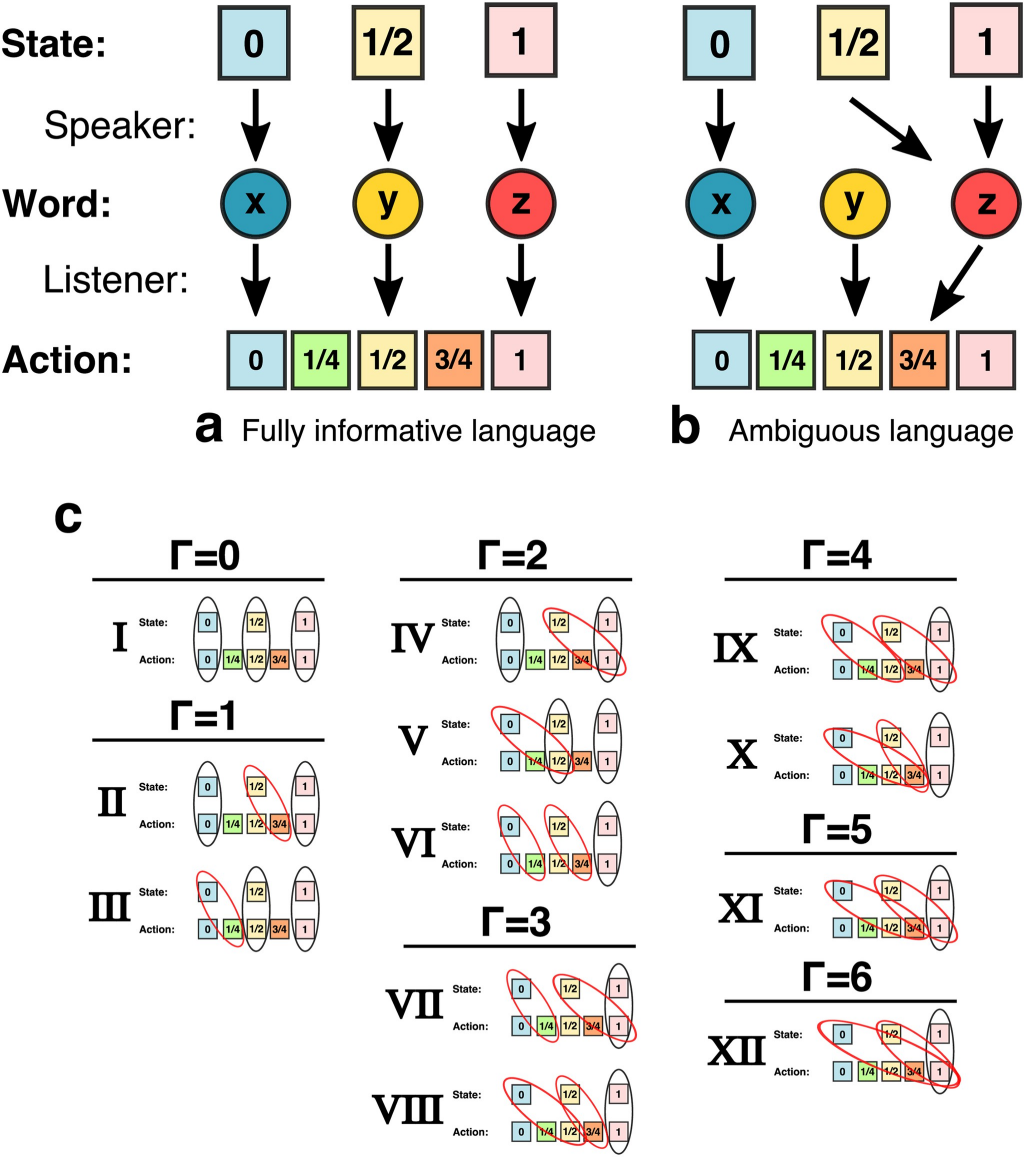
A representation of how misalignment of interests could lead to linguistic change. **(a)**: A fully informative language. The speaker uses a different word for each object, allowing the listener to perfectly translate each word back into the object it came from, and to act optimally in response—that is, to choose an action value equal to the object value. **(b)**: An ambiguous language, using the same polysemic word for objects 1/2 and 1. The polysemy confuses the listener, who cannot translate it accurately, and therefore cannot identify the object when it is 1/2 or 1. **(c)**: For the states and actions shown here, there is one configuration of the speaker’s preferred action per state, $a_{S}^{*}\left(s_{i}\right)$ that is aligned with the listener’s preferred action per state (game **I**), as well as eleven possible scenarios where $a_{S}^{*}\left(s_{i}\right)>s_{i}$ for at least one state; that is, the speaker would prefer an action greater than the listener would prefer. The degree of misalignment for the whole game, Γ, can be graded as the sum of misalignments per state, times the frequency of that state. Here we additionally multiply Γ by 12, simply so the net misalignment is always a whole number. These games are shown in larger scale in Fig B in S1 File.

The optimal action for the listener to take in a given state *s*_*i*_ numerically matches that state—$a_{L}^{*}\left(s_{i}\right)=s_{i}$—so that the listener’s action can be interpreted as her ‘estimate’ of the true state (see S1 File for derivations of speaker and listener optima). The listener action that is optimal for the speaker in a given state, $a_{S}^{*}\left(s_{i}\right)$, can be greater than that which is optimal for the listener, with the disparity between the two optima quantifying the misalignment of interests in this state: $\gamma \left(s_{i}\right)=a_{S}^{*}\left(s_{i}\right)-a_{L}^{*}\left(s_{i}\right)$. We will assume that *γ*(*s*_*i*_) ⩾ 0 ∀*i*; that is, there are no situations in which a speaker would somehow want “less” than dictated by the true state. The overall misalignment of interests in the game is defined as the average disparity between speaker and listener optima, Γ = ∑_*i*_ *p*_*i*_*γ*(*s*_*i*_) (later, when we move to the precise misalignment scenarios depicted graphically in Fig 1 and Fig B in S1 File, we multiply this number by 12, simply so Γ may be written as whole numbers). Given a state *s*_*i*_ and a listener action *a*_*i*_, the listener and speaker’s payoffs decline with the distance of the action from their respective optima. Closing the system therefore requires a choice of a distance function. For instance, Crawford and Sobel [39] employed the distance function *d*(*s*, a*) = (*s* − a*)^2^. In S1 File, we show that many of their key results actually generalize to very broad classes of convex distance functions.

A speaker’s strategy is defined by a speaking matrix **P**, where entry *P*_*ij*_ is the probability that she speaks word *w*_*j*_ upon observing state *s*_*i*_. A listener’s strategy is defined by a matrix **Q**, where *Q*_*jk*_ is the probability that the listener takes action *a*_*k*_ upon hearing word *w*_*j*_. When a speaker with strategy **P** meets a listener with strategy **Q**, their average payoffs are given by

$$\begin{array}{c} \Pi_{S}\left(P,Q\right)=\sum_{i=1}^{I}(p_{i}\sum_{j=1}^{J}\sum_{k=1}^{K}-P_{ij}Q_{jk}d_{S}\left(s_{i},a_{k}\right))=\text{Tr}\left(PQD_{S}\right), \end{array}$$ (1)

$$\begin{array}{c} \Pi_{L}\left(Q,P\right)=\sum_{i=1}^{I}(p_{i}\sum_{j=1}^{J}\sum_{k=1}^{K}-P_{ij}Q_{jk}d_{L}\left(s_{i},a_{k}\right))=\text{Tr}\left(PQD_{L}\right), \end{array}$$ (2)

where Tr denotes the matrix trace and **D**_*S*_ and **D**_*L*_ are matrices encoding the distance functions between the state and the (s)peaker and (l)istener’s optimal actions (these are recorded graphically, in terms of the speaker/listener optimal actions, in Fig 1, and are written out longhand in Table A in S1 File). These equations simply sum up the payoff for each player, assuming that during play, there are many iterations in which the speaker observes a new state drawn with probability *p*_*i*_, says word *w*_*j*_ with probability *P*_*ij*_, to which the listener responds with action *a*_*k*_ with probability *Q*_*jk*_. In the limit of many such iterations, we pass from a stochastic process to a deterministic one: the combination of states, words, and actions will be seen in exact proportions to their relative probabilities. In Fig C(a-b) in S1 File, we graphically work out an example for the set of states, signals, actions, and possible misalignments shown in Fig 1, working from the extensive form of the game (which holds for any misalignment structure, with only the payoffs changing based on which degree of misalignment described in game I-XII is employed) up through the derivation of Eqs (1) and (2).

If **P** and **Q** were restricted to be binary matrices (*P*_*ij*_ ∈ {0, 1}, *Q*_*jk*_ ∈ {0, 1}), then each player would have only a finite number of (pure) strategies available. The game would resemble Crawford-Sobel signalling games [2, 24], with a similar setup having been employed in organizational economics [40, 41]. Here it is known that multiple equilibria exist, which can be quantified by the number of signals used by the speaker—in our case, either three words, two words, or one word. An advantage of our formulation, as well as our evolutionary dynamics described below, is that we can examine all possible mixed states (any real-valued *P*_*ij*_ and *Q*_*jk*_).

### Symmetric play

It is usually assumed [1, 3–9] that a player in a language game can be a speaker and a listener in different situations. We define the *symmetric* version of the game such that when two players meet, they play the game twice, with each player assuming each role (speaker and listener) once. In the symmetric case, a player’s strategy is a speaker-listener matrix pair (**P**, **Q**). Following Eqs (1) and (2), the expected payoff to a player with strategy pair (**P**, **Q**) against a player with strategy pair (**P**′, **Q**′) is

$$\begin{array}{c} \Pi \left(P,Q|P^{\prime },Q^{\prime }\right)=\text{Tr}(PQ^{\prime }D_{S})+\text{Tr}\left(P^{\prime }QD_{L}\right). \end{array}$$ (3)

#### Asymmetric play

The case considered above is that of a symmetric language game, with both players in a given interaction alternately assuming the roles of speaker and listener. Many instances of communication, both in human and non-human communication, are instead best thought of as asymmetric language games, where the two players in an interaction have set roles: one is always the speaker, and the other is always the listener. Consider the example of a bird chick signalling its nutritional need to its mother. The reciprocal interaction does not occur, and so the situation is best modelled as a game where the chick is always the speaker and the mother is always the listener. In the asymmetric case, a speaker and listener’s payoffs from their interaction are given by Eqs (1) and (2) above.

### Evolutionary dynamics

In the symmetric case, we assume that there is a single population of size *N* evolving according to a Wright-Fisher process [42]. Each generation, each player receives their average payoff Π from interacting with every other player, acting once as speaker and once as listener in each interaction. Payoffs are translated to fitness via *f* = exp(*η*Π_*tot*_), where *η* > 0 governs the strength of selection. For each offspring in the next generation, a parent is chosen from the current generation, with probabilities proportional to fitness. Linguistic change arises via imperfect learning and mutation. With probability 1 − *μ*, an offspring learns its parent’s speaker-listener strategy empirically, but does so imperfectly: for each state *s*_*i*_, the offspring samples *k* instances of word choice from the parent’s speaker strategy **P**, and for each word *w*_*j*_, the offspring samples *k* instances of action choice from the parent’s listener strategy **Q**, thus forming its own speaker-listener strategies [43]. With probability *μ*, an offspring ‘mutates’ to a random speaker-listener strategy (**P**, **Q**), with every possible strategy equally likely.

In the asymmetric case, there are two populations, one of speakers and one of listeners, each of size *N*. Each generation, each player receives their average payoff from interacting with every member of the other population. Payoffs Π are translated to fitnesses via *f* = exp(*η*Π_*tot*_), with the strength of selection *η* common to both populations. In each population, parents are again chosen with probabilities proportional to their fitnesses, and each offspring either mutates (with probability *μ* choosing a random strategy—speaker or listener depending on its population) or imperfectly learns its parent’s strategy by sampling it *k* times.

In both cases, we allow the population or populations to evolve for a set number of generations (typically 5, 000—see Methods). The evolutionary dynamics, including the game structure, Wright-Fisher process, and variation induced by mutations and imperfect learning, are graphically worked out for a toy example in Fig C in S1 File.

#### Restricting the state, word, and action spaces

We shall restrict our focus to games with only three words in *W*, for two reasons. First, together with the assumption of small state and action sets, this will make comprehensive analysis of the dynamics computationally feasible. Second, from standard results in the theory of signalling with imperfect alignment of interests, we expect the number of words used in equilibrium languages of a broad class of games to be three or less, except when the misalignment of interests *γ* is extremely small, where full alignment of interests and the accompanying large lexica return (for instance, in Crawford-Sobel-type models there are only three words in equilibria lexica for *γ* > 0.04; we describe and further generalize these results in S1 File). Suppose, therefore, that there are three states *s*_*i*_ ∈ {0, 1/2, 1} (with each observed a third of the time), three available words, *w*_*i*_ ∈ {*x*, *y*, *z*}, and five possible actions, *a*_*i*_ ∈ {0, 1/4, 1/2, 3/4, 1} (Fig 1). The numerical values have been chosen specifically to capture the various possible listener optima, given her uncertainty in the true state induced by ambiguity in the language employed by the speaker.

One can easily check that, for *γ*(*s*_*i*_) ⩾ *s*_*i*_, there are twelve games possible under this restriction, as shown in Fig 1 (in larger scale in Fig B in S1 File). For convenience and simplicity, we assume that each state is equally likely (*p*_1_ = *p*_2_ = *p*_3_), we rescale Γ = 12 ∑_*i*_ *p*_*i*_*γ*(*s*_*i*_) so that Γ only assumes integer values equal to the number of discrete “steps” between the speaker and listener optimal actions, and we define our distance function to simply be the absolute value of the difference between the selected action and each player’s optimal action: $\pi_{L}\left(a_{k}|s_{i}\right)=-|a_{k}-a_{L}^{*}\left(s_{i}\right)\left|=-\right|a_{k}-s_{i}|$ and $\pi_{S}\left(a_{k}|s_{i}\right)=-\left|a_{k}-a_{S}^{*}\left(s_{i}\right)\right|$.

We choose the initial state to be a perfectly informative language in which all speakers employ word *x* for state 0, word *y* for state 1/2, and word *z* for state 1, while all listeners perform action 0 upon hearing word *x*, 1/2 upon hearing *y*, and 1 upon hearing *z*. From this initial state, we allow the population to evolve according to the dynamics described above.

## Results

### Misalignment of interests leads to loss in lexical breadth

**Fig 2:**
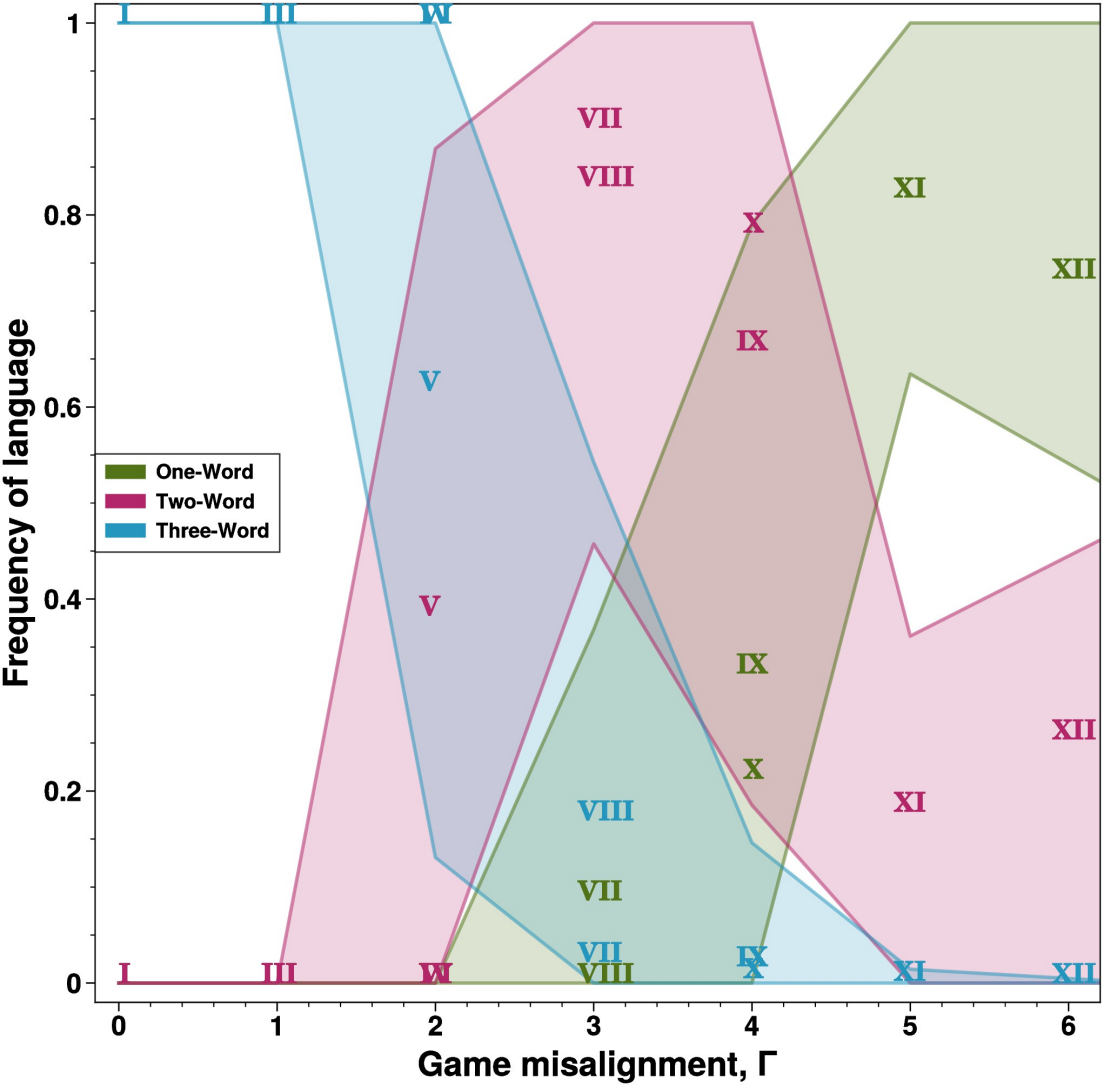
Misalignment of interests leads to loss of lexical breadth in a language. Here we show the average frequency of one-word and two-word languages in the asymmetric game(s) with *k* = 100 and *μ* = 0.001. A one-word language has two columns of *P*_*ij*_ summing to 0 (two words never used), while a two-word language has one column of *P*_*ij*_ summing to 0. Increasing misalignment leads to invasion by two-word languages and then by one-word languages. Roman numerals denote mean values, whereas ribbons denote one standard deviation above/below the mean. Nearly identical results hold for the symmetric case, see Fig E in S1 File. The overlapping game symbols for Γ = 1 are **II,III**, and for Γ = 2 are **IV,VI**.

For each of the twelve games possible under our general setup (Fig 1), Fig 2 displays the average fraction of speakers using one, two, or three words after 1000 generations of evolution, in the case of asymmetric play with *k* = 100 and *μ* = 0.001. It can be seen that, as the degree of speaker-listener misalignment of interests Γ increases, the number of words employed in the most common languages (their lexical breadth) decreases (meaning *P*_*ij*_ = 0 ∀*i* for one or more words *w*_*j*_). The maximum number of words, 3, tend to be used when misalignment is small (Γ < 2), while two words tend to be used for intermediate degrees of misalignment (2 ⩽ Γ ⩽ 4). When misalignment is severe (Γ > 4), perfectly uninformative languages, with only one word, tend to be spoken.

This is consistent with the possible equilibria described by Crawford and Sobel [39], but also shows that the most informative equilibria in the equilibrium sets tend to be used, thereby addressing the *equilibrium selection problem* discussed in the Introduction.

Note that, in some cases, multiple games are associated with the same value of Γ, so that there can be variation in mean outcomes for the same Γ value based on the internal semantics of each game. A prime example is rendered by Game V, the only of the three Γ = 2 games (IV,V,VI) for which we observe two-word equilibria (and indeed observe them quite often) in Fig 2. Despite a passing similarity to Game IV (each map two out of the three states to one single ideal action), the signal usage by the speaker is quite different—there is no scenario in which the speaker would like to signal for an action other than 1/2 or 1. As a consequence, the word natively associated with state *s* = 0 at the beginning of the dynamics is free to associate with either *a* = 1/2 or *a* = 1, and the listener is under selective pressure to ignore this signal entirely while this situation holds.

It is also easy to show that this reduction in lexicon leads to an overall reduction in the information content of the language. For instance, consider the Kullback-Leibler divergence *K*_*w*_(*p*_*L*_(*s*|*w*), *p*(*s*)) = ∑_*i*_ *p*_*L*_(*s*_*i*_|*w*) log (*p*_*L*_(*s*_*i*_|*w*)/*p*(*s*_*i*_)) between the listener’s inferred distribution of states conditioned on receiving a particular signal *w* and the true probability of states. This is a natural measure for the information content of a single signal which has often been used in the literature [9]. It is easy to check that this quantity vanishes when *p*_*L*_(*s*|*w*) matches the true distribution *p*(*s*); that is, when the listener gains absolutely no information about the true state after receiving the signal *w*.

We show an example of the Kullback-Leibler divergence for a game with Γ = 3 in Fig D in S1 File. As expected, misalignment leads to a loss of signal information content, since this is precisely what the speaker desires—she wants to “trick” the listener into giving an action that is closer to her optimal action ${\bar{a}}_{S}$. Notably, it is Word 1, the word originally associated with the state *s* = 0, that loses the most information; this makes intuitive sense, since the speaker will never desire the action *a* = 0 to be played and will therefore be constantly changing their usage of this word to try to elicit a strong response.

The shrinkage of the lexicon due to speaker-listener misalignment is not dependent on the type of play (asymmetric versus symmetric) or the learning parameter *k* (see Figs E, K, and L in S1 File). However, we note that as a function of how we incorporate mutations (a new lexicon is drawn with the usage probability of each word and action picked uniformly-at-random, with the constraint that the probabilities sum to 1), high mutation rates will preserve the original lexical breadth.

### Misalignment of interests leads to cyclical changes in signal meaning

**Fig 3:**
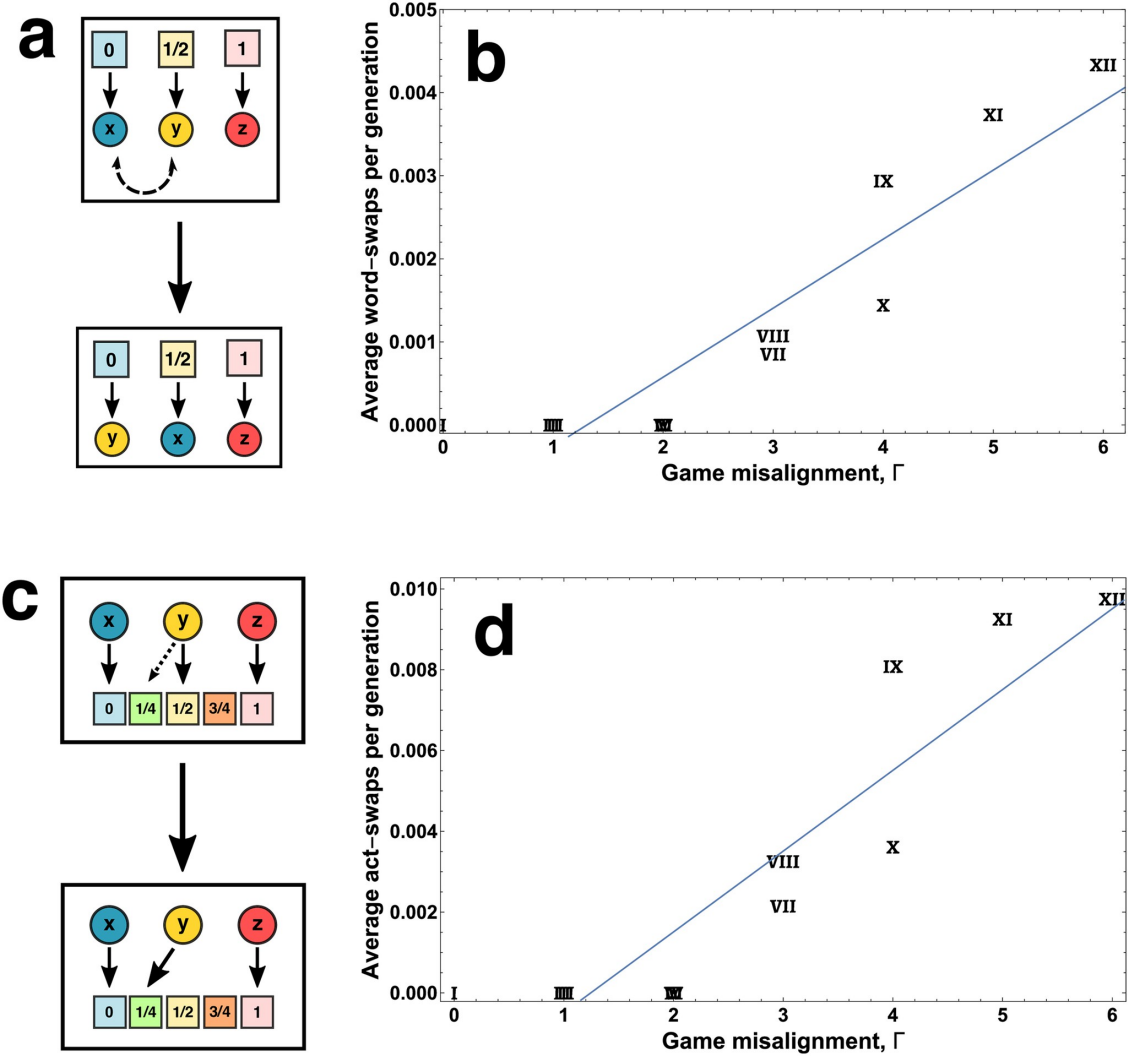
Meanings often change over time due to misaligned interests. (**a**): Changes in the speakers’ perception of their lexica. In each generation, we keep track of whether or not the most probable word used for a state among the population changes (swaps). Tallying the number of swaps gives one measure of change in lexical meaning over time. Note that while a word can be most probable for more than one state, we require a swap lead to changing in the overall strategic *ranking* of available words in the eyes of the population of speakers (**b**): The average number of word-swaps per generation in all twelve games, with *k* = 100 and *μ* = 0.001. The blue line denotes a linear best-fit. (**c**): Changes in the listeners’ perception of their lexica. Similar to (**a**), we tally the number of changes in the most probable action for the listeners in response to a word. However, unlike in (**a**), here we measure simply the steps (in Hamming distance) between the previous and current generation. (**d**): The average number of action swaps per generation in all twelve games, with *k* = 100 and *μ* = 0.001. The blue line denotes a linear best-fit. All panels here are for the asymmetric game, but the symmetric game has nearly identical results, see Fig H in S1 File. The overlapping game symbols for Γ = 1 are **II,III**, and for Γ = 2 are **IV,V,VI**.

In addition to changing the size of the speaker and listener lexica, misalignment can also lead to significant change in the *meaning* or *interpretation* of words by both parties. We can quantify this change by simply tracking the instances of words swapping places in the population-level “ranking” of words in association with states. The ranking is induced by the fact that the states *s*_*i*_ are graded—the word which is spoken with highest probability for state *s*_1_ = 0 is inherently “weaker” (in a supposed psychological sense) than other words at a given point in time, because in every game the speaker’s desired action preserves the ranking of the original states (*γ*(*s*_*i*_) > 0 ∀*i*, so ${\bar{a}}_{S}\left(s_{1}\right)⩽{\bar{a}}_{S}\left(s_{2}\right)⩽{\bar{a}}_{S}\left(s_{3}\right)$). Language change can lead to a re-ranking, where the most probable word for a certain state changes; for instance, the word that was originally associated with a lower state by the speaker can later become associated with a higher state. In terms of basic language use, this can be thought of in some sense as a continuous construction and dissolution of a set of *synonyms*. While the initial language (which is preserved in the absence of misalignment) contains three words with distinct meanings to both speaker and listener, what evolves in the presence of misalignment is a situation in which the speaker is constantly “reappropriating” words associated with lower states to try to elicit a high-state reaction, and often utilizing more than one signal in pursuit of this result. (Fig 3a), and a word that originally elicits a high action by the listener can later elicit a lower action (Fig 3c). We calculated the average number of re-rankings per realization for each game and parameters *k* = 100 and *μ* = 0.001, which we show for the speaker in Fig 3b and for the listener in Fig 3d. For both players, the rate of change of signal meaning correlates well with the misalignment of interests.

These metrics are inherently local, comparing most probable words and actions of the current generation to the previous generation. A global metric of linguistic change can be derived from the fact that we initiate all speakers and listeners with the same, perfectly informative language (Fig 1, panel a). A long-term measure of change is given by comparing the frequency with which a word is used for the same state as it is in the initial language, averaged over the entire population. Normalized so the fraction of “original use” is 1 in the initial language, this can be written as 1 − *S*_*ij*_*δ*_*ji*_/3 (speaker) and $1-L_{ij}\delta_{ji}^{*}/3$ (listener), where $S_{ij}=\sum_{n}P_{ij}^{\left(n\right)}/N$ and $L_{ij}=\sum_{n}P_{ij}^{\left(n\right)}/N$, where *N* is the population size and Einstein summation conventions are used. *δ*_*ij*_ is the identity 3 × 3 matrix, whereas $\delta_{ij}^{*}$ is the identity 3 × 5 matrix. These metrics are therefore the trace of the average speaker and listener matrix (written thus because the trace on the non-square matrix *L*_*ij*_ is otherwise ill-defined)—this trace is the probability of the population (on average) using (or interpreting) the word *x* for state *s*_1_, *y* for *s*_2_, and *z* for *s*_3_, which was the initial language.

**Fig 4:**
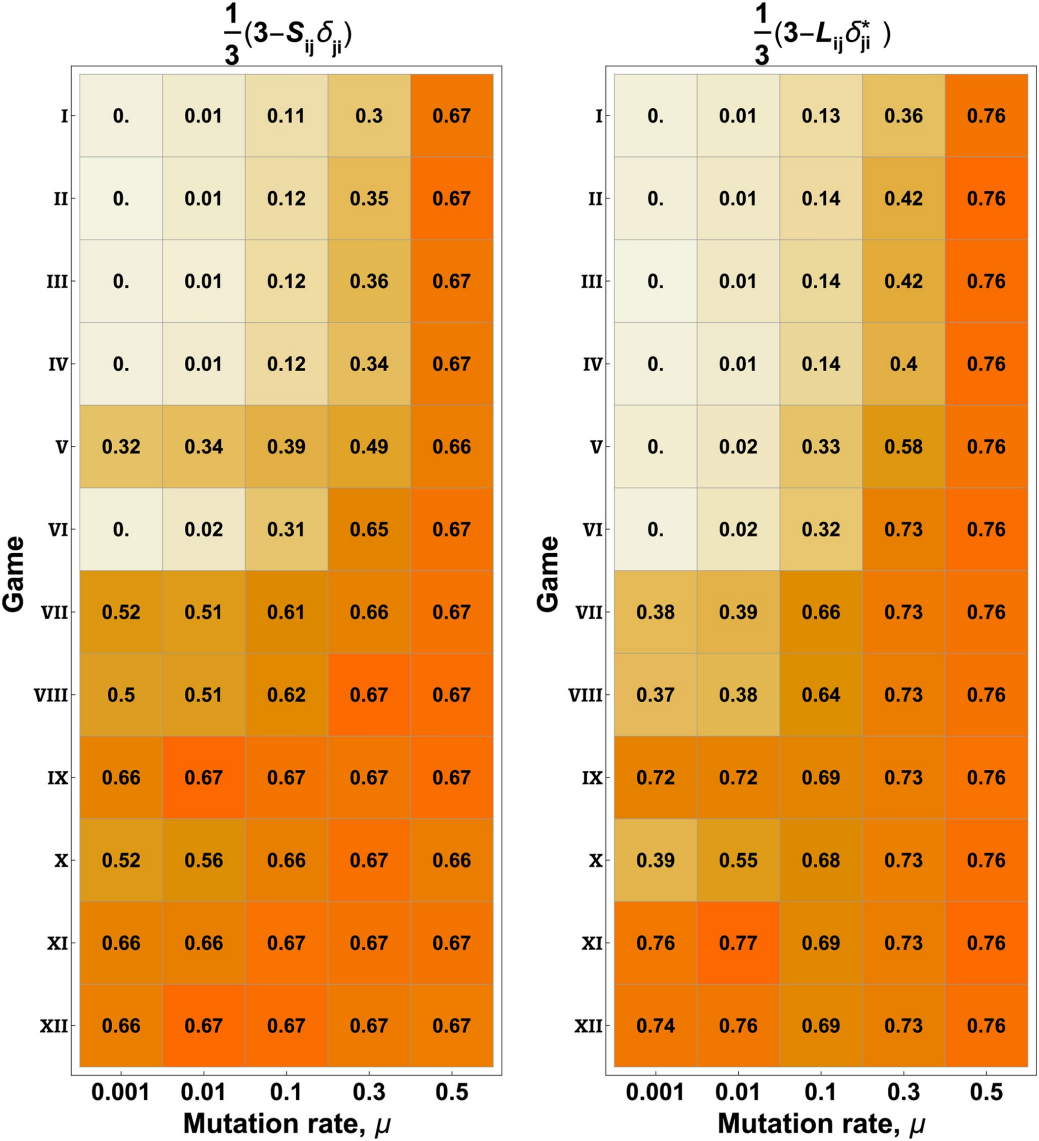
Original meaning of words is lost with increasing misalignment. (**left**): A natural measure for long-term language change is with what probability speakers use word *x* for state 0, *y* for 1/2, and *z* for 1 at the end of the game, since this is the fully-informative language which constitutes the initial condition for all players. This can be written as a “percentage change” in the form 1 − (*S*_*ij*_*δ*_*ji*_)/3, where $S_{ij}=\sum_{n}P_{ij}^{\left(n\right)}/N$ is the population-averaged speaking matrix and $L_{ij}=\sum_{n}Q_{ij}^{\left(n\right)}/N$ is the population-averaged listening matrix (and Einstein summation conventions have been implemented). (**Right**): A similar metric for the listeners. $\delta_{ii}^{*}$ is understood to be the 3 × 5 matrix with $\delta_{1,1}^{*}=\delta_{2,3}^{*}=\delta_{3,5}^{*}=1$ and all other entries equal to zero. Both panels shown are for the asymmetric game, however the symmetric game shows nearly identical results, see Fig I in S1 File. Note that the upper bounds of 2/3 (left) and 4/5 (right) reflect two different situations—in the limit of high *μ*, each word becomes equiprobable for each state (probability 1/3 for speakers, so **Tr**(**S**) = 1, and 1/5 for listeners, so $L_{ij}\delta_{ji}^{*}=3/5$); in the limit of high Γ, one-word languages come to dominate (Fig 2), so one diagonal element of **S** is always equal to 1 and the rest to 0.

We measured these global changes from language use in the original language for all twelve games and various mutation rates, averaging the metrics over many realizations (Fig 4). In the high-mutation limit, it is obvious that the original language should be completely lost, since here approximately half the population samples an entirely new language in every generation. This makes a good point of comparison in the low-mutation limit, where sufficient misalignment leads to the same values. Note, however, that the actual linguistic situation is very different between these two cases—in the high-mutation limit, a word becomes equiprobable for all states and vice-versa, so having three states and three words means each word is associated with its original state 1/3 of the time. In the low-mutation, high-misalignment limit, only one word is used for all states, and so that word is also used with its original meaning 1/3 of the time. The numerical equivalence of how much meaning is retained in these two limits illustrates the fact that misalignment of interests can evolve a population whose language is just as unintelligible to the original population as complete scrambling of the language (babelization). Furthermore, these results are robust against the type of play (asymmetric versus symmetric) and the value of the learning parameter *k* employed (S1 File).

### The evolutionary importance of novel words

We next allowed for the introduction of new words into the repertoire. In real language, words with a severe literal meaning are often co-opted to become hyperbolic, such as *to starve* (from a Germanic root *sterben* meaning “to die”). This is in line with the discussion of dishonest hyperbole in the Introduction—the user of the word is attempting to elicit some kind of strong response out of the listener by using a word which can also be associated with a factually-dire situation. We therefore assume that, whenever a new word is introduced, it begins with the most extreme possible interpretation by the listener. To examine the effect of regularly introducing such new words, we considered a version of the game where, after every 500 generations, a new word is introduced into the lexicon (in other words, a new column in the speaker matrix and a new row in the listener matrix). Speakers learn to use the new word because mutation introduces a new lexicon which with probability 1 uses the new word in association with one of the three states.

It is not clear *a priori* what the effect of routinely introducing new words should be. Our earlier results on lexical shrinkage with increasing misalignment would seem to suggest that some signals (though not necessarily the freshly-introduced ones) should be routinely lost, in order to preserve the lexicon size that is under dynamic selection. However, the injection of new available words essentially pushes the system far-from-equilibrium, so that it is no longer obvious that we are simulating the equilibrium selection problem for a Crawford-Sobel type model; if new words appear faster than the listener can “learn to ignore them”, it expands the speaker’s ability to misdirect and trick the listener by shuffling the intended meanings of the words. Finally, introducing new signals should reduce the overall frequency of most signals (since the frequencies must sum to 1)—in the context of regular lexical language, this would suggest that some infrequent words should regularly disappear simply due to fluctuations.

**Fig 5:**
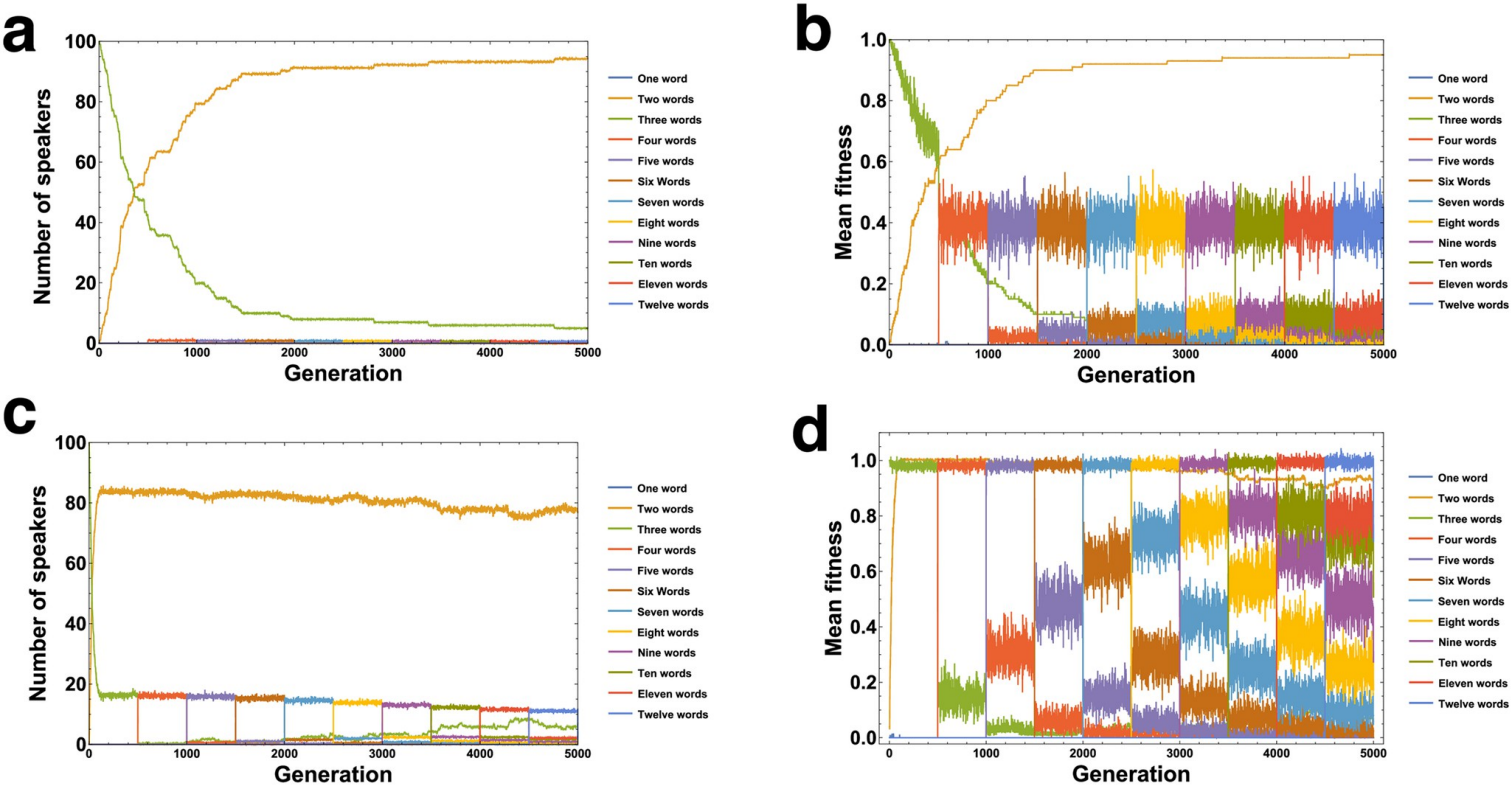
Mutation rate (innovation) leads to preservation of old intensifiers, as well as “verbose” individuals (those who use all possible words). Here we show averaged results over 100 realizations in the asymmetric version of Game **V** with *k* = 10 and *μ* = 0.005 (**a,b**) versus *μ* = 0.1 (**c,d**) In this rendition, a new word becomes available via de-lexicalization every 500 generations, beginning from our normal three words in the first generation. (**a**): The average number of speakers using *n* ≠ 2 words exists at an extremely small mutation-selection balance in comparison to two-word speakers. (**b**): The mean fitness of verbose individuals is always dominated by that of two-word speakers. (**c**): At higher mutation rates, verbose speakers are found in a higher proportion roughly matching the ratio of the two mutation rates. (**d**): However, their mean fitness is much higher than in (**b**), and the most verbose speakers in any period are equally fit, and always dominate less verbose *n* ≠ 2 speakers.

In fact, allowing for the constant innovation of new hyperbolic terms leads to a surprising result—the restoration of lexica that use every available word. This is in stark contrast to our earlier results, where misalignment could cause some words to be completely lost from the language. For instance, Game **V** shows a strong bias towards two-word languages (Figs 1 and 2) in the absence of new words. When new words arrive regularly, however, many speakers are found to use all available words (even the new words), and these “verbose” speakers (the speakers who use all available words) can even be more fit than the “terse” speakers (who use even fewer than the initial set of words) which are advantageous when new words are not added. This depends on the mutation rate, which in the context of innovation describes how quickly a new term is taken up by the population once it appears. The importance of this rate can be understood at least intuitively in terms of the dynamic frequency-dependent fitnesses of words. When the mutation rate (or, the rate of innovation of invidual lexica) is low, the “terse” two-word language and even the native three-word language exist at higher frequencies than new, broader lexica (Fig 5a and 5b).

**Fig 6:**
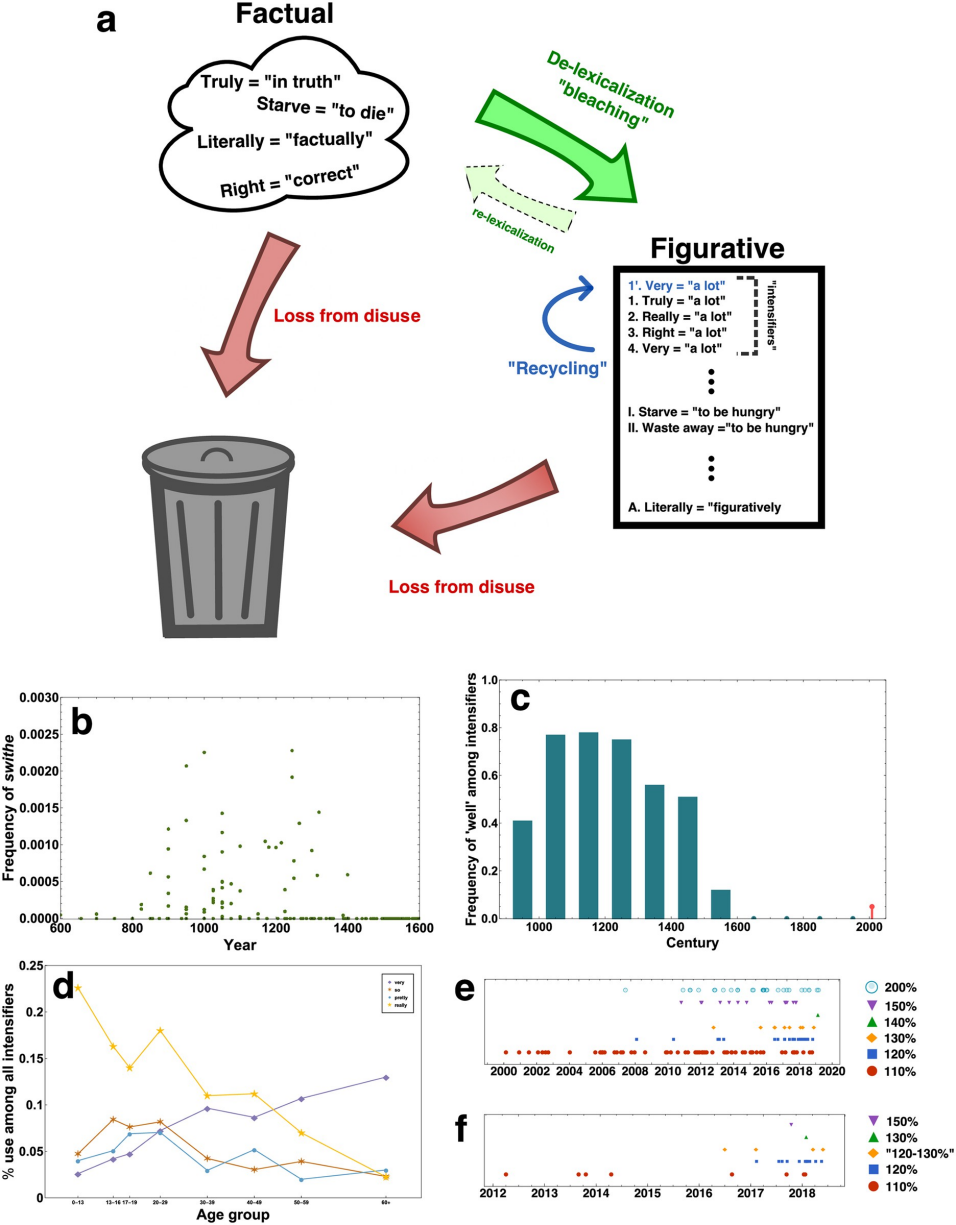
The life cycle of intensifiers, with empirical examples. (**a**): Most hyperbolic language originates as factual, indicative language that is then re-purposed (“de-lexicalization”, shown in green) as a figurative device. Once a word has “used up” its hyperbolic value, it decreases in usage and is supplanted either by a new bleached word or, frequently, by an older hyperbolic term whose loss of value has been forgotten by the current generation (a process called “recycling”, shown in blue). When words fall below a certain threshold of use without being recycled, they are lost from the language (red). (**b**): The rise and fall of *swithe*, the original English intensifer, from its de-lexicalization from the adjective *swith* meaning “strong” through its peak in early Middle English until its loss from the language. Data drawn from a large corpus of poetry and prose [54]. (**c**): The “revival” of intensifying *well*. Teal bars show the average usage of *well* as an intensifier among competing intensifiers from the 10th to the 17th century in the same corpus as (**b**). The red point represents the television show *The Inbetweeners*, where its social context of use may represent a much longer vernacular use not reflected in the literary record [55]. (**d**): Recycling in a particular speech community. The frequency of usage of the four main intensifiers in Toronto when grouped by age of speakers shows that different intensifiers dominate in different sub-communities, with terms unused by an older group (such as *really*) being recycled into the dominant intensifier by a younger generation [51]. (**e**): A novel example of de-lexicalization. We collected instances of football players “giving X% effort” from eight major British periodicals, where *X* is a number greater than 100. The numbers 110, 120, 130, and 140 are introduced in sequence and increase in frequency thereafter, though all continue to be used once they have been introduced. (**f**): A sub-corpus of (**e**) focusing on a single coach (Antonio Conte). We show that the population-level trends are often driven by verbose, innovative individuals, who also preserve older intensifiers at low frequencies in the lexicon.

It is not surprising that, at higher mutation rates, “verbose” individuals with broader lexica persist at much higher frequencies than at low mutation rates, since mutation leads to individuals who use all available words with probability 1. However, what is surprising is the relative fitness of different lexica in this limit (panel (**d**)). Not only are verbose individuals just as fit as terse individuals in this regime, but the hierarchy of verbose individuals is strict—if there are ten words available, then an individual using all ten words is always fitter than an individual using nine words, continuing all the way down to the original three-word language. This is because the way in which words are interpreted by listeners are also changing at a higher rate in this regime, and abandoning a word is equivalent to abandoning a possibly-large payoff from certain listeners when hearing that word. This may partially explain why hyperbolic language is often led by a select group of “leaders” (for instance, compare Fig 6e and 6f), which has been seen in other sociolinguistic contexts as well [44].

### Empirical connections to intensifiers

The model under examination, as well as the results discussed in the previous sections, may seem at first glance to have little bearing on actual language. The fact that for the vast majority of parameter space a “relevant” or “equilibrium” language can consist of only three dominant signals does not comport with most of human experience; in English, even object categories such as nearly-indiscernible colors will consist of more than three synonyms, and quantitatively graded language (such as accompanies size, for instance) can easily enumerate more than a hundred signals, which are used with a more-or-less consistent frequency distribution over time. The tendency for infrequent signals to be under positive selection, and frequent signals to be under negative selection, is also extremely unusual; empirically, the opposite is observed for most of language [45]. As a consequence, the relative ranking (by usage) of words is quite stable over time, having fluctuations which are sub-linear in the current ranking [46].

However, *intensifiers* (in Modern English, some prominent examples being *very, really, pretty, quite*, and *so*) form a broad grammatical class which demonstrate many of these properties. The life cycle of intensifiers is as follows (pictured graphically, with empirical examples, in Fig 6). Intensifiers are rarely (if ever) created *ex nihilo*, but are adapted from normal words with normal meanings through a process of “de-lexicalization” (Fig 6a) [47]. A freshly de-lexicalized intensifier is, over time, used in a wider and wider context, until it becomes usable in nearly any linguistic context, boosting any adjective; as a consequence, it is often supposed that its “surprisal” value diminishes with overuse (Fig 6b) [48, 49]. At this point, in contrast to normal words, its frequency diminishes, so that the most-used intensifier is consistently being replaced. Consistent with the idea of selection based on “surprisal”, low-ranking intensifiers tend to make a comeback; this volatility in the usage ranking is called “recycling” in the linguistics community [50, 51] (interestingly, similar dynamics of word genesis are seen in the language of fashion [52, 53]). As a consequence, intensifiers tend to persist even as the repertoire gets wider and wider, rather than dying out (for instance in Fig 6, panels e and f). Some intensifiers can even reappear in certain speech communities after exceptionally long periods without a recorded usage (Fig 6c). Some intensifiers seem to have completely died out (for instance, no English reader is likely to recognize *swithe*, the earliest recorded English intensifier, whose dynamics in a particular written corpus are shown in Fig 6b).

The concept of “surprisal” in association with these terms is crucial, also, in connecting intensifiers to the motivations of our model. Firstly, in connection with the theory of ranking dynamics, it strongly suggests that intensifiers have frequency-dependent fitness [46], as is seen with signals in our model. However, it also suggests a psychological motivation which is consistent with dishonest hyperbole, and therefore also with misaligned interests.

To strengthen these associations, we compared eight studies on intensifiers (Fig F in S1 File) and analyzed their re-rankings in the same manner as we have for the signal dynamics in our games (Fig G in S1 File). There are a few notable similarities to our toy model. Firstly, each study tracks no more than five intensifiers; while it should be supposed that other intensifiers exist within the studied speech community at low frequencies (waiting to be recycled into usage), the number of signals is consistent with our games. Secondly, the rate of re-ranking shares quantitative overlap with our games (in Fig 3, the rates range from 0.0 to 0.01; in Fig G in S1 File, from 0.002 to 0.051). Finally, the re-ranking rates might suggest consistency with the psychological explanation via “surprisal”—written corpora have very low rates of re-ranking, whereas a study focused exclusively on teenage speech has the highest rate of re-ranking by a large margin.

Finally, to draw especial parallels between the quantitative nature of the game and intensifiers, we also generated our own unique dataset. We collected examples from British football periodicals over the past twenty years of individuals “giving X% effort on the pitch”, where *X* is a number greater than the physically-realizable *X* = 100 (Fig 6e). The numbers *X* = 110, 120, 130, 140 are introduced in succession, with each one appearing roughly around the same time the previous one becomes quite frequent—showing both “de-lexicalization” and “recycling”. We focus on a particularly verbose individual in Fig 6f, to highlight the connection with the “verbose” subpopulation of speakers in our games with expanding available lexica.

## Discussion

The study of signalling is a wide and interdisciplinary field with deep connections to linguistics, biology, sociology, economics and beyond. Both the linguistics literature (including relevant schools of pragmatics such as Grice [56, 57], Horn [58], and the “relevance” school [59]) as well as the game-theoretic literature [1, 3–9, 30–34], often assume perfect alignment of the goals of speaker and listener, a situation which leads to maximum information flow. This is undoubtedly the most common situation in signalling, even when hyperbolic signals (signals which communicate a state more extreme than the actual state) are used.

It is not uncommon, however, for the speaker and listener to have misaligned interests. Chick begging, hormonal signaling, job applications, company valuations and dating sites all represent cases from the fields above where the speaker may want to signal “a little more” than the factual state in order to get a more favorable response from the listener (signaling more hunger to get additional food, for instance). The equilibria of games representing such signaling situations were originally examined by Crawford and Sobel [2], with further analysis (and experimental examination [60]) of these equilibria suggesting which one might be selected in the infinite-time, infinite-population case [61–63]. Casting this game in an evolutionary dynamics context can shed light on this issue. The breadth of theoretical work suggesting which equilibrium might be selected in a dynamic context speaks to the inherent volatility of the game. This is perhaps best understood intuitively by one of the proposed theoretical equilibrium-selection thresholds, NITS (no incentive to separate) [63]. The NITS claim is that an equilibrium is only selected if it is advantageous over “honesty”, that is, the perfectly-aligned language. However, this is strongly dependent on the speaker state. High states (strong job applicants, hungry birds) would prefer everyone to use an honest language; low states benefit from dishonesty. The conflicting interest between these parties, generation after generation, is what leads to the rich and rather unique dynamics of these games.

Here we employed a toy model with a small number of states, words, and actions which cover a wide range of Nash equilibria for a wide range of models of utility functions for speaker and listener and amounts of misalignment between speaker and listener. However, it is not exhaustive. For decreasing rates of misalignment, larger and larger lexica become possible. We suspect that in those regimes, the dynamics we see here are not relevant; in Figs 2–4, the “unusual” dynamics only begin for Γ ⩾ 2, so at rates of misalignment small enough to support larger lexica, results from classic language and signaling games with aligned interest probably apply. Crucially, however, these limitations on lexicon size are simply from considerations of static Nash equilibria. Initially, we expected our evolutionary game to demonstrate which equilibrium was selected in a dynamical process by an evolving population—indeed, this is what is seen in the first results section. Even in the final results section, where more words are periodically introduced into the system, the Crawford-Sobel results seem to be supported at small mutation rates, because these lexica quickly die out in favor of the maximum lexicon size predicted to be a viable Nash equilibrium. However, at higher mutation rates, something unpredictable happens from the perspective of the static equilibrium analysis in the literature–verbose individuals (using arbitrarily many signals) become at least as fit as the predicted terse equilibrium, and persist (albeit at low frequency). This may suggest that there are other interesting phenomena that occur in these games far-from-equilibrium, suggesting future work studying the dynamics could be very fruitful. We furthermore emphasize that, while the key assumptions in the Crawford-Sobel class of models are difficult to relax, there are other sub-classes that do not fall under the umbrella we discuss here. In S1 File, we have replicated parts of our analysis for a class of models that incorporate misalignment as a multiplicative rather than additive factor. Our results suggest that these two classes are deeply similar, and we believe our toy model covers a wide variety of instances that could be drawn from either the additive or multiplicative classes. However, it is not impossible that more complex models could be amenable to larger lexicon size, or have dynamics that differ in a qualitatively different way. Furthermore, we do not mean to argue that the larger lexica which occur for very small values of misalignment are unimportant. This transition regime, while rather narrow in the amount of misalignment represented, could be rich in interesting and unusual dynamics, and is likely also of comparable if not greater relevance to human language, since in the empirical datasets we present throughout the number of intensifiers is usually greater than three (though, it is to be emphasized, never substantially larger).

On the linguistic side of things, we believe the most common example of misaligned signalling to be a type of hyperbolic language called intensifiers, a grammatical category of words which boost the value of an adjective [36, 37]—words like *so, very, quite*, and so forth. Intensifiers are often studied because of their unique and interesting dynamics. They are generated (“de-lexicalized”) from words which originally have a stark yet factual meaning, such as the oldest recorded English intensifier, *swithe*, those original Old English lexicalized adjective form simply meant “strong” (see also our example in Fig 6, where each new football hyperbole is quantitatively greater than the one preceding it). We believe this to be a clear illustration of their purpose, to demand attention via their hyperbolic nature. This is further supported by their second unusual dynamic, “recycling”—the propensity of rarely-used intensifiers to become common in the future (the idea being that by becoming rare, they have “recharged” their hyperbolic value), rather than to go extinct. In other words, the future ranking of intensifiers is *anti*-correlated with its current ranking, unlike most linguistic items (and rankings in general [46]) where the future ranking is either independent of or correlated with the current ranking.

Our work extends the game-theoretic aspect of misaligned signalling and additionally connects it to the linguistic literature via an analysis of intensifiers. While some game-theoretic studies [2, 10, 61–66] have considered misalignment of interest, none of these works focus on the actual evolutionary trajectories of the words themselves, or on the dynamics of word re-rankings over time. We use an evolutionary model, which can distinguish which of the (multiple) equilibria in the above studies is actually selected by a finite population, an important disambiguation. Additionally, in order to demonstrate the deeper connections between the linguistic and game-theoretic aspects of signalling studies, we have explicitly tracked the dynamics of the words. As a result, we are able to show that misalignment alone is sufficient to explain the unique and interesting dynamics behind intensifiers, such as de-lexicalization and recycling.

In particular, we have demonstrated that misalignment can have dramatic effects on the size and internal ranking of signal lexica in a population—depending on the degree of misalignment and the rate at which new intensifiers enter the language, (de-lexicalization) the lexicon of an individual is under selective pressure to shrink (low rate of de-lexicalization) or rapidly expand (high rate of de-lexicalization), with little room for intermediate strategies. In addition, when the size of an individual’s lexicon is not changing, there are still rich dynamics in the form of *recycling*, or re-ranking of signals by a speaker in terms of usage and by a listener in terms of magnitude of appropriate response. Both our analysis of empirical data and the results of our simulations suggest that the rate of recycling is proportional to the immediate misalignment of interests. While it is not explicitly part of our model, a reasonable interpretation is that the greatest listener response comes from words with the greatest “novelty”—listeners rapidly become accustomed to hearing a certain word in a certain situation and are therefore under pressure to discount it, allowing for an improbable word in that same situation to earn a much greater response.

The different degrees of misalignment in our games could be interpreted in many different ways, but it may be more realistic to interpret them as different social situations, perhaps representing different amounts of “immediacy”: an individual in a job interview has higher immediacy than a writer of fiction who will never meet their reader, for instance. This would be parsimonious with the empirical results we collected suggesting that the rates of recycling are lowest in written texts and highest in teenage speech (Fig F in S1 File)—while we have limited corpora showing diachronic change in teenage speech, this intuition is further supported by the observation in other studies that the number of intensifiers in the average teenage lexicon is at least twice that of the average adult [67, 68].

Our work suggests many extensions and novel ideas in the sociolinguistic and signalling literature. It is very likely that there are other common categories of hyperbolic, misaligned signaling to which our modeling framework should be immediately applicable. Misalignment is also not a single fixed quantity in a population—different individuals can have different degrees of misalignment, something we have not investigated in this study. The distribution of such values within a population, as well as the possibility of population structure, suggest multiple possible theoretical extensions. The connection between the social axis (measured by the degree of misalignment) and the temporal axis (measured by the rate of recycling) is also intriguing and may have extensions in other domains, such as sound change, which is known to have a heavily social dynamics [44]. This also suggests the possibility of larger-scale empirical study to either confirm or vitiate a correlation between bulk social factors and rates of linguistic change.

## Methods

### Simulations

Each member of the initial population, of size 200 (symmetric game) or size 100 (asymmetric game, meaning 100 each of speakers and listeners, so the number of players is still 200), begins with the speaking matrix **P** = *δ*_*ij*_, the identity matrix (so that word *x* is used for state *s*_1_ = 0, word *y* for state *s*_2_ = 1/2, and word *z* for word *s*_3_ = 1 with 100% probability), and listening matrix $Q=\delta_{ij}^{*}$, the 3 × 5 matrix with $\delta_{1,1}^{*}=1$, $\delta_{2,3}^{*}=1$, $\delta_{3,5}^{*}=1$, and all other entries zero (so that word *x* leads to action *a*_1_ = 0, word *y* to *a*_3_ = 1/2, and word *z* to *a*_5_ = 1 with 100% probability). Therefore, in the absence of any changes in **P** and **Q**, the perfectly informative game will be played by all individuals *ad infinitum*.

However, changes are incorporated at the individual level via imperfect learning, as well as mutation. After each round of play (during which every player plays the game against every other player in the population), the parental distribution of the next generation is chosen according to a Wright-Fisher process: for a given offspring, the probability that its parent is a given member of the previous generation is proportional to that given member’s fitness in its own generation. Note that, due to the linear properties of the trace operator, the fitness for each individual can be calculated very quickly from Eqs (1) and (2)—the sum of traces is the trace of the sum, so for instance in the asymmetric game, the total payoff for a speaker with strategy **P** after playing every listener with strategy **Q**_*n*_ is $\sum_{n=1}^{100}\text{Tr}\left(PQ_{n}D_{S}\right)=\text{Tr}\left(P\left(\sum_{n=1}^{100}Q_{n}\right)D_{S}\right)$. Once the parents of the members of the next generation have been decided, offspring sample their parents’ speaking and listening matrices according to the ‘parental learning’ method [4], with learning parameter *k*. This means that in **P**, for each object, an offspring draws *k* words from its parent’s distribution over words for that object. These *k* draws define the offspring’s speaking distribution over words for that object. A similar procedure holds for **Q**. The lower *k* is, the less accurately the offspring learns the distribution of its parents, which leads to change once certain words and actions are not played with 100% probability. This initial change, then, arises via random mutations. After parental learning, each offspring has a chance of having its speaking matrix mutate with probability *μ*, and (independently) a chance of having its listening matrix mutate with probability *μ* (in the asymmetric game, there is only room for one possible mutation per player). In either case, the mutant matrix is chosen from a uniform distribution over the appropriate matrix space. A mutant, then, samples an entirely different usage of the words at hand.

The payoffs according to which parents are chosen are specified by Eq (3) for the symmetric game or Eqs (1) and (2). The payoffs Π are transformed to positive fitnesses by the exponential transformation *f* = *exp*(*η*Π), where *η* = 10 is the strength of selection (this strength of selection might appear large, but recall that the payoffs of the game are small in magnitude).

A realization consists of the process described above, repeated for 5, 000 generations. This number is arbitrary, as mutation-selection dynamics continue without cessation; however, for most values of *k* and *μ*, this is sufficient generations for our metrics to reach steady-state mutation-selection balance; see, for instance, Fig K in S1 File. We ran both the symmetric and asymmetric versions of the game for the twelve games described in Fig 1 (in larger size in Fig B in S1 File) and the parameters *k* ∈ [10, 100, 1000, 10000] and *μ* ∈ [0.001, 0.01, 0.1, 0.3, 0.5], each for 1, 000 realizations. Most plots in the main text represent summary statistics (means and standard deviations) for fixed games and parameters over all realizations at those values.

### Data for Fig 6

Frequency counts of *swithe* (Panel A) and *well* (Panel B) are drawn from a corpus of literature over the course of the English language based on the Penn Corpus of Historical English, the Parsed Corpus of Middle English Poetry, and Project Gutenberg; details of this corpus are discussed in a separate manuscript [54]. Data on *well* in *The Inbetweeners* (Panel B), as well as on intensifier usage by age group in Toronto (Panel C), were taken from prior publications [51, 55].

To collect the data on football journalism used in panels D and E, we used a Google Custom Search Engine (search engine ID: 007380079963265108005:jhnywllqxqg) combined with the native JSON API, which was aimed particularly at the football sections of eight major British periodicals: the *BBC*, *The Sun*, the *Telegraph*, the *Daily Mail*, the *Independent*, the *Mirror*, the *Express*, and *The Guardian*. We collected all instances of [110, 120, 130, 140, 150, 200, 300, 500, 1000]% effort, where “%” could employ either the sign or the word(s) “percent” or “per cent”. Next, Unix regular expression functions were used to ensure that if two articles appeared within 7 days of one another with the same number phrase and the same coach mentioned, the second (and third, fourth, etc.) instance of the article was omitted, in order to eliminate the (common) phenomenon in which the same hyperbolic utterance given once at a press conference was repeated multiple times among the different news outlets. The number of remaining instances was small enough (< 300 in total) such that the set could be manually curated to check for any further duplicates, as well as instances where the percentages referred to either a non-salient feature (such as increases in a team’s budget, say) or a player’s health (“Player X is not injured, but rather at 110 per cent”) which we took to be nonrelevant to this study.

Links to articles with the dates provided in Fig 6e are given in Supplementary Information File 2. Links to articles with the dates provided in Fig 6f are given in Supplementary Information File 3.

## Supporting information

S1 File Supporting analysis and figures. (PDF)

S2 File Dates and hyperlinks for Fig 6e. (TXT)

S3 File Dates and hyperlinks for Fig 6f. (TXT)
