# Supplementary material for: Evolutionary dynamics of hyperbolic language
Source: PLoS Comput Biol. 2023 Feb 23;19(2):e1010872. doi: 10.1371/journal.pcbi.1010872 (PMC9949666; doi:10.1371/journal.pcbi.1010872)
Supplement: S1 File — (PDF) [file pcbi.1010872.s001.pdf]

Supplementary Information for: Evolutionary dynamics of hyperbolic language

Madison S. Krieger[1,*]

[1]Organismic and Evolutionary Biology, Harvard University, Cambridge, Massachusetts, United States of America

[*]To whom correspondence may be addressed. Email: mkrieger@fas.harvard.edu.

Discussion of Crawford-Sobel-type models

In their paper, Crawford and Sobel [1] derive very general results on (Bayesian) Nash equilibria and statics for a signaling game with the assumptions: (i) there are two players, a speaker and a listener, with $(\mathbf{C})^2$ utility functions $U^S(a, s, \gamma)$ and $U^L(a, s)$, where a is the listener's action, s is the state observed only by the speaker, and γ is a measure of misalignment between the state and the speaker's optimal action taken by the listener; (ii) U^S and U^L have a unique maximum in a for any tuple (s, γ); (iii) $\frac{\partial^2}{\partial a \partial s} U^i(\cdot) > 0$, where i indicates S or L (this condition ensures that, with full information, the optimal action $\bar{a}_i$ for speaker and listener is monotonic increasing in s. It is straightforward to write

$$\bar{a}_L(x, y) = argmax \int_x^y U^L(a, s)\zeta(s)ds, \tag{1}$$

where ζ_s is the distribution of states. This simply means that, on an interval between two states $y > x$, the listener's best option is the option that maximizes their utility function, given the distribution of states between x and y. $\bar{a}_S$ depends on how the misalignment γ enters the speaker's utility function.

In their Theorem 1, they prove four critical facts: (1) for any γ such that $\bar{a}_S(s, \gamma) \neq \bar{a}_L(s)\ \forall s$, there is an integer $N(\gamma)$ such that every n with $1 \leqslant n \leqslant N(\gamma)$ is the number of signals in at least one equilibrium; (2) there are no other equilibria (i.e. $N(\gamma)$ is the maximum number of possible words/signals in an equilibrium); (3) each equilibrium is *separating*, meaning that signals by the speaker are assigned to nonoverlapping intervals of states (bounded therefore by discrete endpoints $\{s_0, s_1, s_2, ...\}$, and the listener's action is uniquely defined by that word/interval of states; (4) on the boundaries between these intervals, the speaker should be insensitive to the different values of the listener's chosen action on both sides of the boundaries. This last "arbitrage" condition can be written as

$$U^S(\bar{a}_L(s_i, s_{i+1}), s_i, \gamma) = U^S(\bar{a}_L(s_{i-1}, s_i), s_i, \gamma). \tag{2}$$

In a specific toy example, they consider the exact utility functions $U^S(a, s, \gamma) = -(a - (s + \gamma))^2$ and $U^L = -(a - s)^2$, with $\zeta(s)$ the uniform distribution. Without loss of generality, they map all possible states onto the unit interval: $s \in [0, 1]$. We will follow their analysis of the optimal actions and maximum number of words in equilbria, but instead we will expand the utility functions considered to two classes: $U^S(a, s, \gamma) = -(a - (s + \gamma))^n$ and $U^L = -(a - s)^n$ (with n even, else the underlying assumptions cannot be met); $U^S(a, s, \gamma) = -e^{-(a-(s+\gamma))^n}$ and $U^L = -e^{-(a-s)^n}$ (again with n even). These cover a broad class of models which fit the underlying assumptions and treat misalignment as additive.

We first calculate the optimal action for the listener on a given partition. Equation S1 can be written as

$$\frac{\partial}{\partial a} \int_{s_i}^{s_{i+1}} U^L(a, s)ds = 0, \tag{3}$$

since we assume $\zeta(s)$ is the uniform distribution. Note that in our considered classes of U^L, a and s are symmetric. So it is easy to simplify this, for all these classes, as

$$U^L(a, s_i) - U^L(a, s_{i+1}) = 0. \tag{4}$$

It is now clear that the powers of n (and whether or not the model is exponential or polynomial) will cancel when solving the above for $\bar{a}_L(s_i, s_{i+1})$. Indeed, one can check via direct calculation that for all n in either class, $\bar{a}_L(s_i, s_{i+1}) = \frac{s_i+s_{i+1}}{2}$, as seen in the Crawford-Sobel paper. This is also the justification for why we include five actions in our model. Three actions (0,1/2,1) are the Nash equilibria in the absence of misalignment (which is one of our twelve models). The other two (1/4, 3/4) are the Nash equilibria in the presence of misalignment.

The maximum number of possible words in an equilibrium comes from examining the arbitrage condition (equation S2), now that we know $\bar{a}_L(s_i, s_{i+1})$, and making sure it is soluble for a given fixed value of γ. The arbitrage condition has a certain symmetry, which we write out for the polynomial class:

$$-\left(\frac{s_i + s_{i+1}}{2} - s_i - \gamma\right)^n = -\left(\frac{s_{i-1} + s_i}{2} - s_i - \gamma\right)^n. \tag{5}$$

It should be clear that in both the polynomial and exponential cases it is easy to resolve ("cancel out") the exponentiation and powers of n, assuming we desire only real-valued solutions. Since the s_i are monotonically increasing, this relationship can only be satisfied so long as

$$s_{i+1} = 2s_i - s_{i-1} + 4\gamma, \tag{6}$$

which has solution

$$s_i = s_0 + is_1 + 2i(i-1)\gamma. \tag{7}$$

Given that $s_0 = 0$ and the last state has $s_N = 1$, there can be no states with $i > N$ such that the rightmost term is bigger than 1. Therefore, the maximum number of partitions (since the speaker says one word per partition, this is equal to the maximum number of words) in a Nash equilibrium in any of these class of models with misalignment γ is

$$N(\gamma) = \lceil -\frac{1}{2} + \frac{1}{2}\left(1 + \frac{2}{\gamma}\right)^{1/2} \rceil \tag{8}$$

where $\lceil\cdot\rceil$ is the ceiling function. This is the relationship plotted in Fig A.

An alternative model with multiplicative misalignment

The previous derivation suggests that, for a wide class of models with additive misalignment, the likely number of words in equilibrium is equivalent to that found by Crawford and Sobel [2]; we therefore think that our toy model is probably representative of a broad number of useful models. However, we can attempt to extend the applicability and generality of our results by also considering a toy model with *multiplicative* misalignment. In this case, rather than $\bar{a}_S = s + \gamma$, we say that $\bar{a}_S = \gamma s$. Note that, besides the mathematical difference, this could reasonably be said to represent a different psychological model for the speaker. In the additive case, the speaker's greed is independent of their state – the size of their lie is held constant. However, in the multiplicative model, the larger the state, the larger the lie becomes. When the state is interpreted as hunger, for instance, the multiplicative model almost represents a sort of "panic". The more dire the situation, the more the speaker amplifies their signal, perhaps in a bid to secure a stockpile of food for future famines. As written, γ could be arbitrarily large – for convenience in plotting and some calculations, it is easier to consider the quantity $\xi = 1/\gamma$, which we will call the alignment of interests, in contrast to the misalignment.

In this section we demonstrate that while this change in model can cause some quantitative differences, the qualitative behavior of the system is still the same in terms of the number of words that can be expected in an equilibrium, as well as the shrinkage of lexicon size with increasing misalignment such that a real evolving population always picks out the Crawford-Sobel (Bayesian) Nash equilibrium with the largest possible vocabulary for that value of misalignment. We interpret these results as further evidence for the generality of our toy model and analysis from the main text, despite their foundation upon a computationally tractable discrete model.

We begin by showing that in a broad class of multiplicative models, the number of words expected in equilibrium is still a small handful (so that the three-word toy model of the main text retains its applicability), comparable to the additive class of models. Note that Eqns. S1-S4 depend only on the utility function of the listener, which is independent of whether the speaker implements their misalignment of interest in an additive or multiplicative manner. The structure of the arbitrage condition (Eqn S5) is unchanged (though, on both sides of the equation, the replacement $(-s_i - \gamma) \to (-\gamma s_i)$ must be made), showing that the number of words in equilibrium is unaffected by particular forms of the utility function, similar to the additive models. The equivalent of Eqn. S6 is

$$s_{i+1} = (4\gamma - 2)s_i - s_{i-1}, \tag{9}$$

which has the solution (equivalent of Eqn S7) of

$$s_i = \frac{s_1}{4\sqrt{\gamma(\gamma-1)}} \left(\left(2\gamma - 1 - 2\sqrt{\gamma(\gamma-1)}\right)^i - \left(2\gamma - 1 + 2\sqrt{\gamma(\gamma-1)}\right)^i \right). \tag{10}$$

This is much more cumbersome than the easily-soluble Eqn S7. However, we can numerically solve for the boundary $s_N = 1$ simply by computing the right-hand side for a fine mesh of values of γ and N, locating the discriminating curve where the right-hand side crosses 1. This function $N(\gamma)$ is shown in Fig M. Similar to the additive case, an infinite number of words can be supported as the players' interests become more and more aligned; however, this number drops incredibly quickly (by $\xi = 1/\gamma = 0.92$, we have already gone from an infinite number of possible words to 5, and by $\xi = 0.61$, $N(\xi) = 2$).

The fact that most languages in both the additive and multiplicative classes of models contain only one, two, or three words already suggests that the toy model discussed in the main text (i.e., one of the twelve games shown in S1 Fig B) is probably a good fit for almost any situation involving communication with misalignment of interests. However, we also simulated the population dynamics, following the same rules as the additive model from the main text. While we did not analyze the dynamics of individual signals with the same level of detail, we did track the fraction of the population which utilized a three-, two-, or one-word language, similar to Fig 2 and S1 Fig D. We present the results for two different values of the mutation rate μ and $k = 100$ in S1 Fig M. The results are very similar to that seen in the main text. Since the distribution of equilibrium max-words $N(\gamma)$ and the loss of lexical breadth with increasing misalignment are qualitatively identical (and indeed quantitatively quite similar) in both broad classes of models, we strongly suspect the dynamics of individual words are similar between the two as well.

As a final example of the utility and generality of this model, we draw attention to a parallel between this multiplicative model of misalignment of interests and Godfray's classic model of offspring begging [3], which might be considered a variant of the Sir Philip Sidney game [4]. We assume that the same graded states in the main-text (and multiplicative) model represent hunger levels of an offspring (a chick, say), and the actions represent the amount of feeding from the chick's mother. The Godfray game differs from our present multiplicative model in that the benefit to the chick is a direct fitness gain of $a \cdot s$, whereas there is a cost to the mother of feeding an amount a, because her reproductive potential will be reduced by $a^2/2$. These forms of the fitness are chosen in particular so that the mother's fitness, which combines that of the focal chick and her future reproductive potential, is $as - a^2/2$, which is maximized at $a = s$. At this point the Godfray game begins to resemble our games, because the chick inherently would like to be fed an amount $a > s$, whereas the mother is trying to feed an amount satisfying $a = s$, despite misguiding signals from the chick.

Consider now the chick's fitness as a function of its siblings. Following standard treatments of parent-offspring conflict [5] and relatedness [6], we define a 'relatedness' parameter r, which has a value of 1 for the focal chick, 1/2 for a full sibling, and smaller values for more distant siblings. We assume a type of kin selection in which the fitness cost to the focal chick of seeing their sibling fed decreases as their relatedness increases; likewise, that their fitness increase in being overfed decreases as more related siblings go hungry. The focal chick's payoff can be written as $as - rd^2/2$, which is now maximized at $a = s/r$. While the payoff functions are slightly different, this game now strongly resembles our multiplicative games: the speaker (chick) prefers $\bar{a}_S = s/r$ (cf $\bar{a}_S = s/\xi$ for the multiplicative game), whereas in both games the listener (mother) prefers $\bar{a}_L = s$. In fact, the two have very similar evolutionary dynamics as well. Using techniques previously developed [7], we exactly solved both this Godfray game and our asymmetric multiplicative game for a finite population of 100 players with pure strategies and no mutations (S1 Fig O). The population dynamics between the two models are nearly identical, especially away from the boundaries of transition between three, two, and one-word languages.

Supplementary Figures

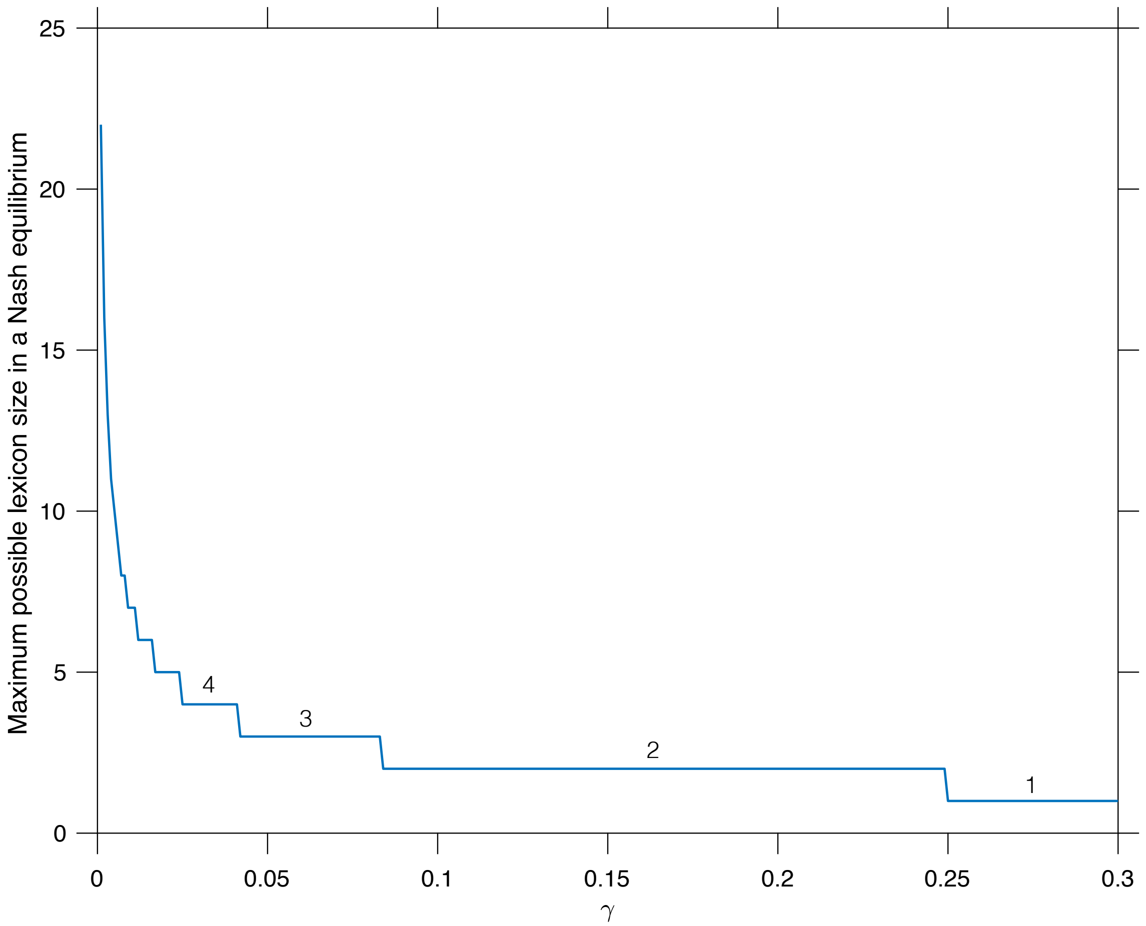

S1 Figure A. The maximum possible number of words in any Nash equilibrium language for the Crawford-Sobel class of models, versus the misalignment parameter γ.

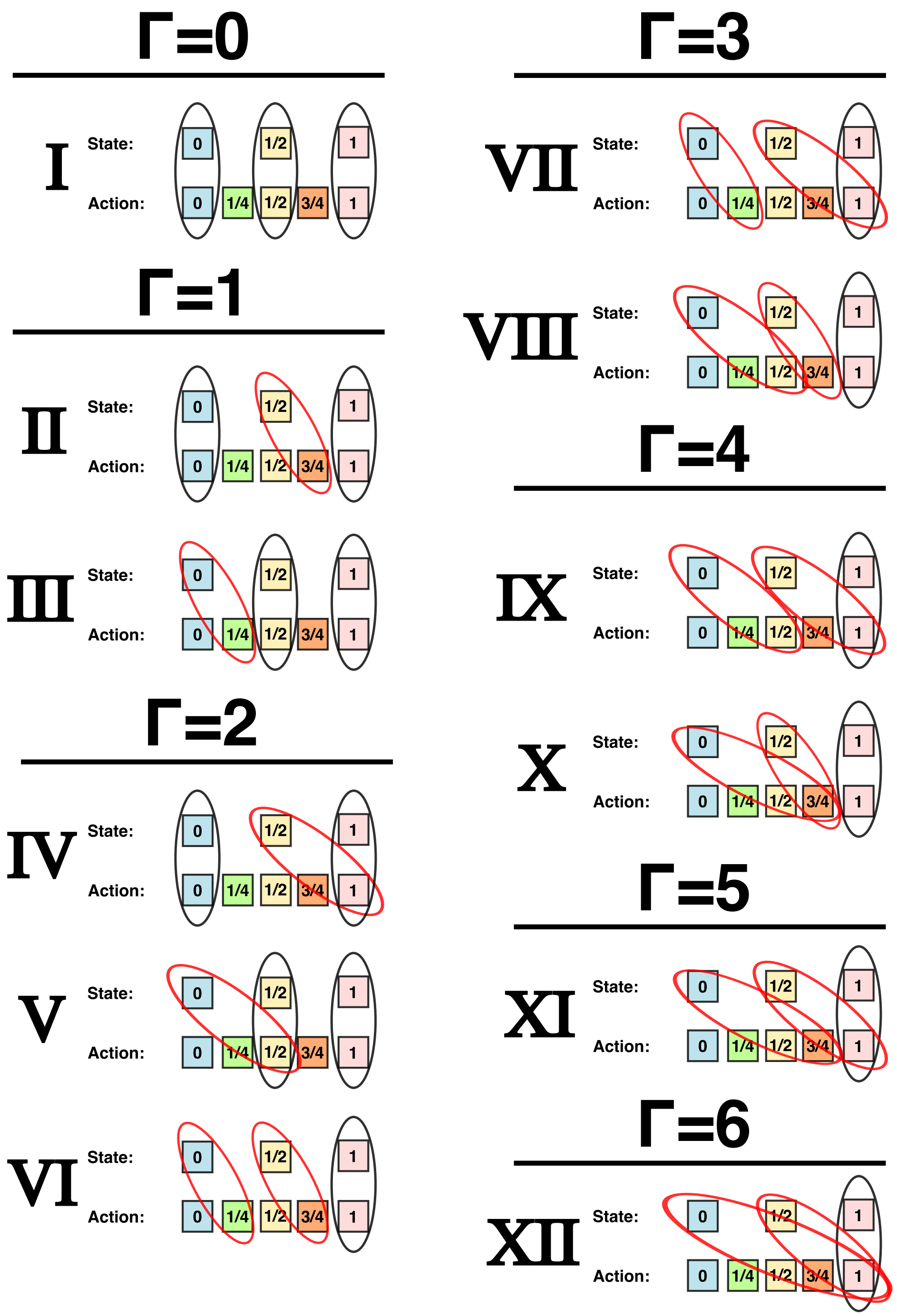

S1 Figure B. Games used to represent misalignment of interests. As a simple representation of how a speaker and listener can have misaligned interests, we considered all possible games in the setup shown in Fig 1 where the speaker can have an optimal response from the listener that differs from the actual state observed by the speaker. The listener always wants to give the action that corresponds to the state observed by the speaker, and is penalized by the stepwise distance between the action and the state when she fails to do so (i.e., the number of steps along the bottom row between the actions she chose and the actual state). The speaker is penalized by the distance between the action taken by the listener and their *preferred* action, which is prescribed by the game. The misalignment of interests between speaker and listener when the speaker observes state s_i can be measured by γ_i, which is the stepwise distance between the state and the speaker's desired action. Requiring that the speaker can only "want a little more" (not less), and requiring that the speaker's desired actions preserve the ranking of the actual states, leads to twelve possible games which can be listed according to their total misalignment $\Gamma = \sum \gamma_i$. Here, black circles highlight states for which speaker and listener are aligned on what action the listener should take ($\gamma_i = 0$), whereas red circles highlight states when the speaker would prefer to receive "a little more" from the listener than what is warranted by that state ($\gamma_i \neq 0$).

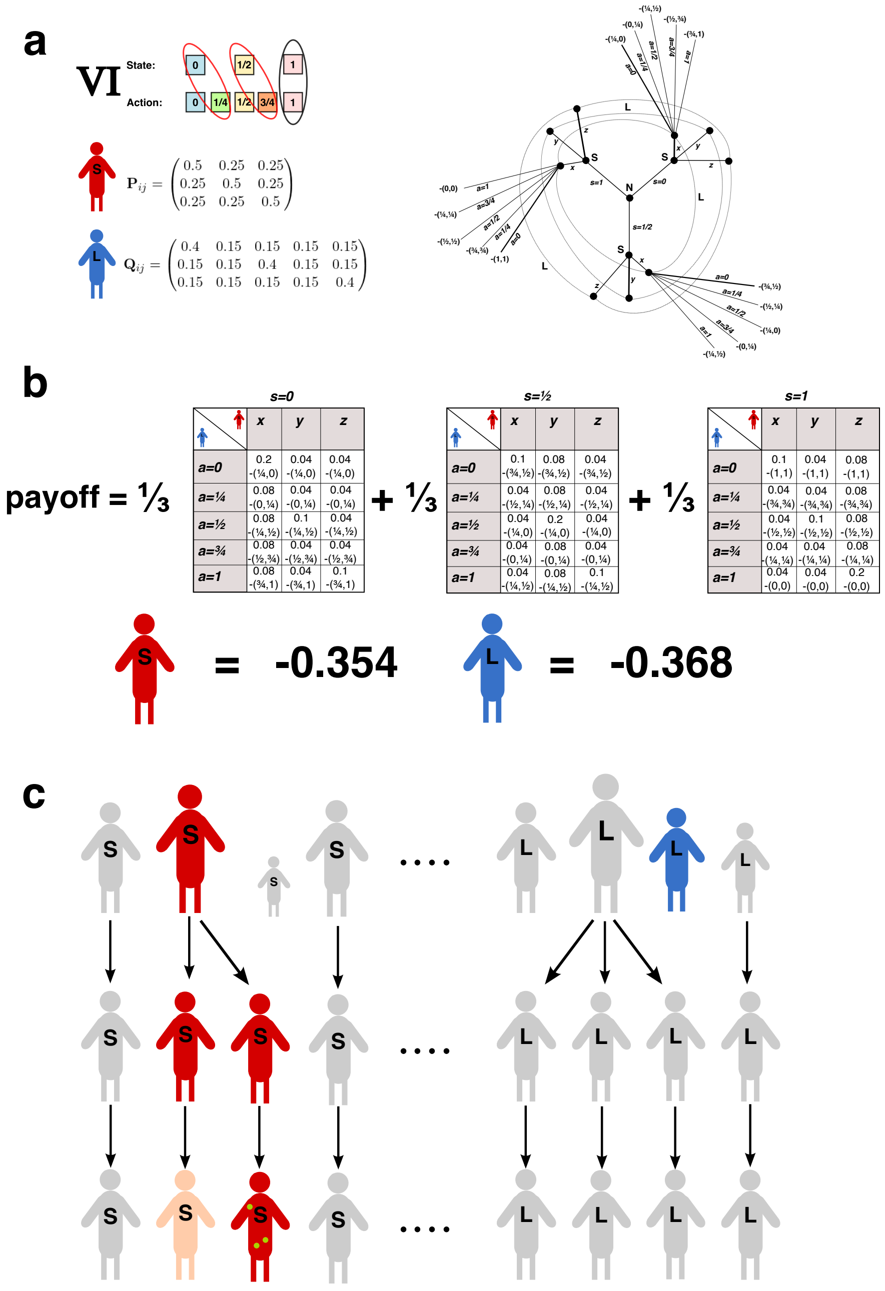

S1 Figure C. A worked example of the model with asymmetric play. (a): We chose two random focal individuals, as well as one game (Game VI) to follow through one round of the evolutionary dynamics. The extensive form of the game is given on the right; edges have thicknesses drawn proportional to the strategies of the focal individuals. Dotted lines represent information sets; the speaker knows the true state, whereas the listener only knows the signal they receive from the speaker. Terminal nodes are only drawn from one of the signals, but importantly, payoffs are never signal-dependent; were the other sets of terminal nodes drawn, only the relative thicknesses (proportional to $\mathbf{Q}_{ij}$) would change. Changing the game to another of those pictured in Fig S2 only changes the payoffs at the terminal nodes (which are written out longhand in S1 Table 1). Payoffs are written in the order (speaker, listener). **(b):** In one round, it is assumed that every individual plays every other individual many times – this eliminates the stochasticity from having mixed strategies. For our two focal players, we can explicitly write the expected normal form of the game after many iterations, which is equivalent to Equations (1) and (2). In these table entries, the top number indicates the probability of that event ($\mathbf{P}_{ij}\mathbf{Q}_{jk}$), whereas the bottom tuple indicates the payoffs for (speaker, listener). **(c):** After our focal players have completed step **(b)** with every other player in the population, fitnesses are assigned according to aggregate payoff. Here, individuals have been re-sized according to fitness. In the first step of the evolutionary dynamic (the Wright-Fisher process), the next generation picks its strategy from the current generation, weighted by fitness (figure size). The second step of the evolutionary dynamic highlights mutation and imperfection in learning. With probability μ, a player picks a new strategy uniformly-at-random (red player becomes orange). There is still variation in the absence of mutations, as offspring draw their strategies from sampling their parent's strategy k times (green speckles).

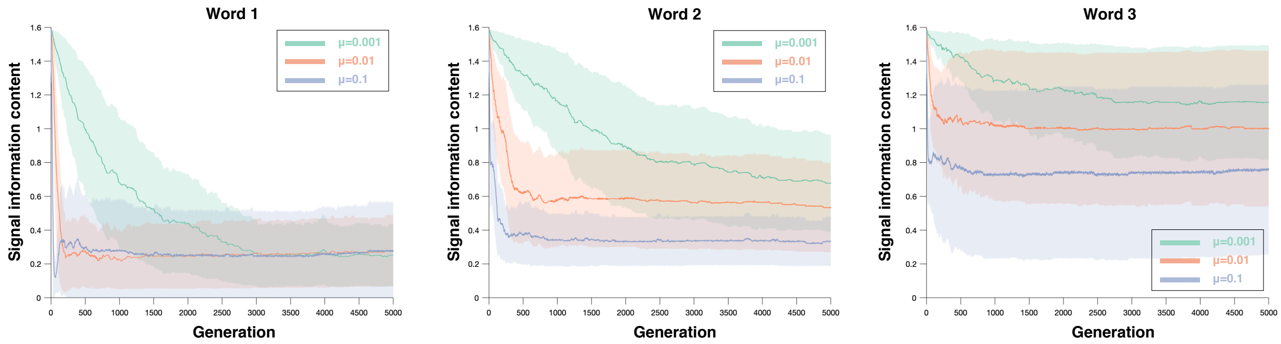

S1 Figure D. Kullback-Leibler divergence shows information loss due to lexicon shrinking. Here we plot the Kullback-Leibler divergence for the three original words of the lexicon for 5,000 realizations of the symmetric Game VIII ($\Gamma = 3$), with $k = 100$ and three values of μ. Lines show the mean values over all realizations, and shaded bands show ± 1 standard deviation from the mean. While the information content of all three words decreases with time, the original Word 1 (which is often lost from the vocabulary in this game) very quickly loses most of its content.

$$\mathbf{D}_L = \begin{bmatrix} 0 & 1/4 & 1/2 & 3/4 & 1 \\ 1/2 & 1/4 & 0 & 1/4 & 1/2 \\ 1 & 3/4 & 1/2 & 1/4 & 0 \end{bmatrix}$$

Game	$\mathbf{D}_S$	Game	$\mathbf{D}_S$
I	$\begin{bmatrix} 0 & 1/4 & 1/2 & 3/4 & 1 \\ 1/2 & 1/4 & 0 & 1/4 & 1/2 \\ 1 & 3/4 & 1/2 & 1/4 & 0 \end{bmatrix}$	VII	$\begin{bmatrix} 1/4 & 0 & 1/4 & 1/2 & 1 \\ 1 & 3/4 & 1/2 & 1/4 & 0 \\ 1 & 3/4 & 1/2 & 1/4 & 0 \end{bmatrix}$
II	$\begin{bmatrix} 0 & 1/4 & 1/2 & 3/4 & 1 \\ 3/4 & 1/2 & 1/4 & 0 & 1/4 \\ 1 & 3/4 & 1/2 & 1/4 & 0 \end{bmatrix}$	VIII	$\begin{bmatrix} 1/2 & 1/4 & 0 & 1/4 & 1/2 \\ 3/4 & 1/2 & 1/4 & 0 & 1/4 \\ 1 & 3/4 & 1/2 & 1/4 & 0 \end{bmatrix}$
III	$\begin{bmatrix} 1/4 & 0 & 1/4 & 1/2 & 3/4 \\ 1/2 & 1/4 & 0 & 1/4 & 1/2 \\ 1 & 3/4 & 1/2 & 1/4 & 0 \end{bmatrix}$	IX	$\begin{bmatrix} 1/2 & 1/4 & 0 & 1/4 & 1/2 \\ 1 & 3/4 & 1/2 & 1/4 & 0 \\ 1 & 3/4 & 1/2 & 1/4 & 0 \end{bmatrix}$
IV	$\begin{bmatrix} 0 & 1/4 & 1/2 & 3/4 & 1 \\ 1 & 3/4 & 1/2 & 1/4 & 0 \\ 1 & 3/4 & 1/2 & 1/4 & 0 \end{bmatrix}$	X	$\begin{bmatrix} 3/4 & 1/2 & 1/4 & 0 & 1/4 \\ 3/4 & 1/2 & 1/4 & 0 & 1/4 \\ 1 & 3/4 & 1/2 & 1/4 & 0 \end{bmatrix}$
V	$\begin{bmatrix} 1/2 & 1/4 & 0 & 1/4 & 1/2 \\ 1/2 & 1/4 & 0 & 1/4 & 1/2 \\ 1 & 3/4 & 1/2 & 1/4 & 0 \end{bmatrix}$	XI	$\begin{bmatrix} 3/4 & 1/2 & 1/4 & 0 & 1/4 \\ 1 & 3/4 & 1/2 & 1/4 & 0 \\ 1 & 3/4 & 1/2 & 1/4 & 0 \end{bmatrix}$
VI	$\begin{bmatrix} 1/4 & 0 & 1/4 & 1/2 & 3/4 \\ 3/4 & 1/2 & 1/4 & 0 & 1/4 \\ 1 & 3/4 & 1/2 & 1/4 & 0 \end{bmatrix}$	XII	$\begin{bmatrix} 1 & 3/4 & 1/2 & 1/4 & 0 \\ 1 & 3/4 & 1/2 & 1/4 & 0 \\ 1 & 3/4 & 1/2 & 1/4 & 0 \end{bmatrix}$

S1 Table A. Speaker ($\mathbf{D}_S$) and listener ($\mathbf{D}_L$) matrices for games I-XII. Distances $\gamma = |s_i - a_k^*|$, where a^* indicates the preferred outcome for the (s)peaker and (l)istener, are shown graphically in Fig1 and S1 Fig A, and are consolidated in matrices where the three rows correspond to the states $s \in \{0, 1/2, 1\}$ and the five columns correspond to the listener's actions $a \in \{0, 1/4, 1/2, 3/4, 1\}$. The listener always prefers the action to correspond to the true state (unseen to her), and therefore has one fixed distance matrix given above the table, whereas the speaker has different preferred outcomes, corresponding to matrices which induce different degrees of misalignmentwith the listener.

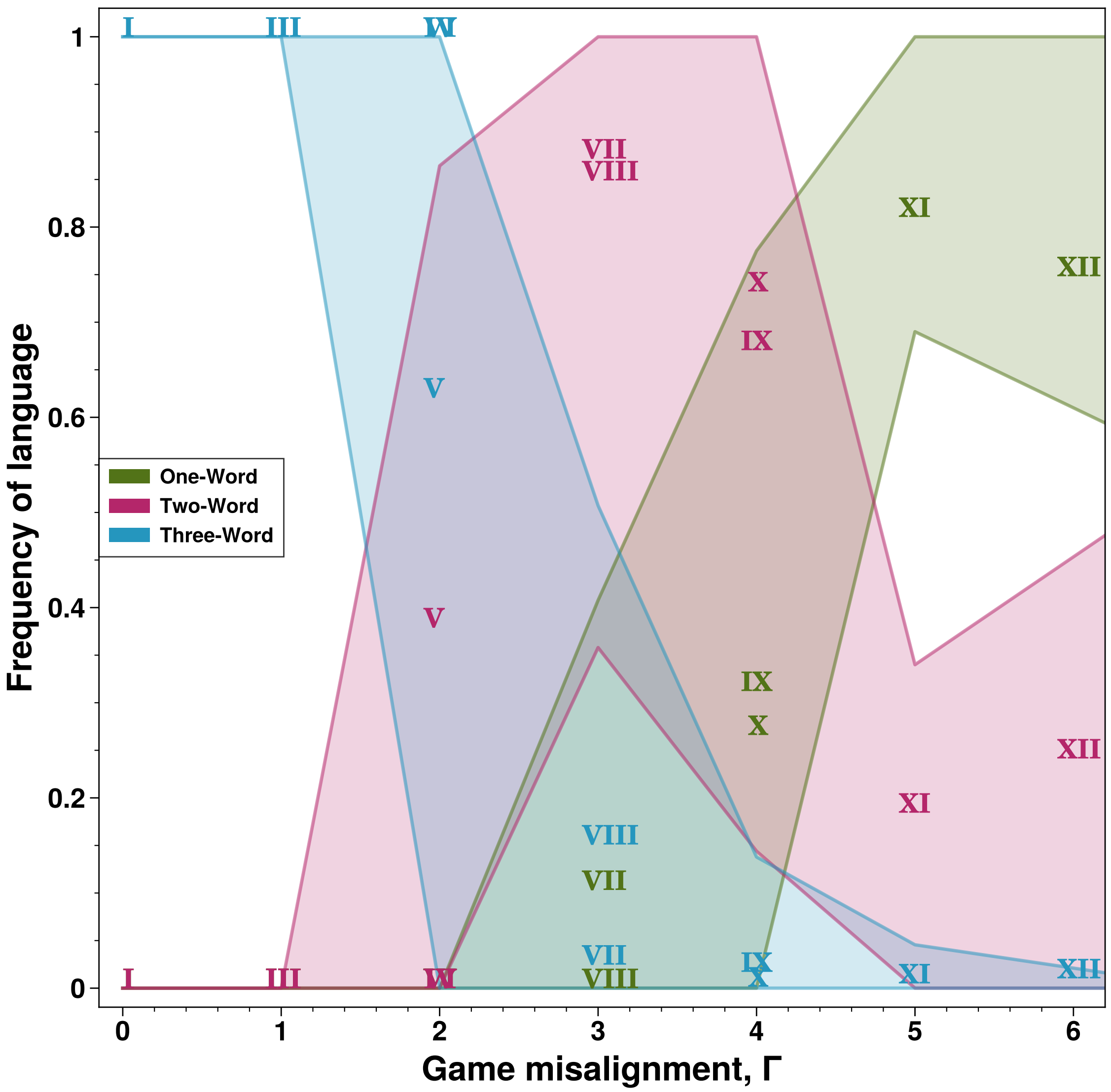

S1 Figure E. Misalignment of interests leads to loss of lexical breadth in a language: The equivalent of Fig 2 for the symmetric game(s). The overlapping game symbols for $\Gamma = 1$ are **II,III**, and for $\Gamma = 2$ are **IV,VI**.

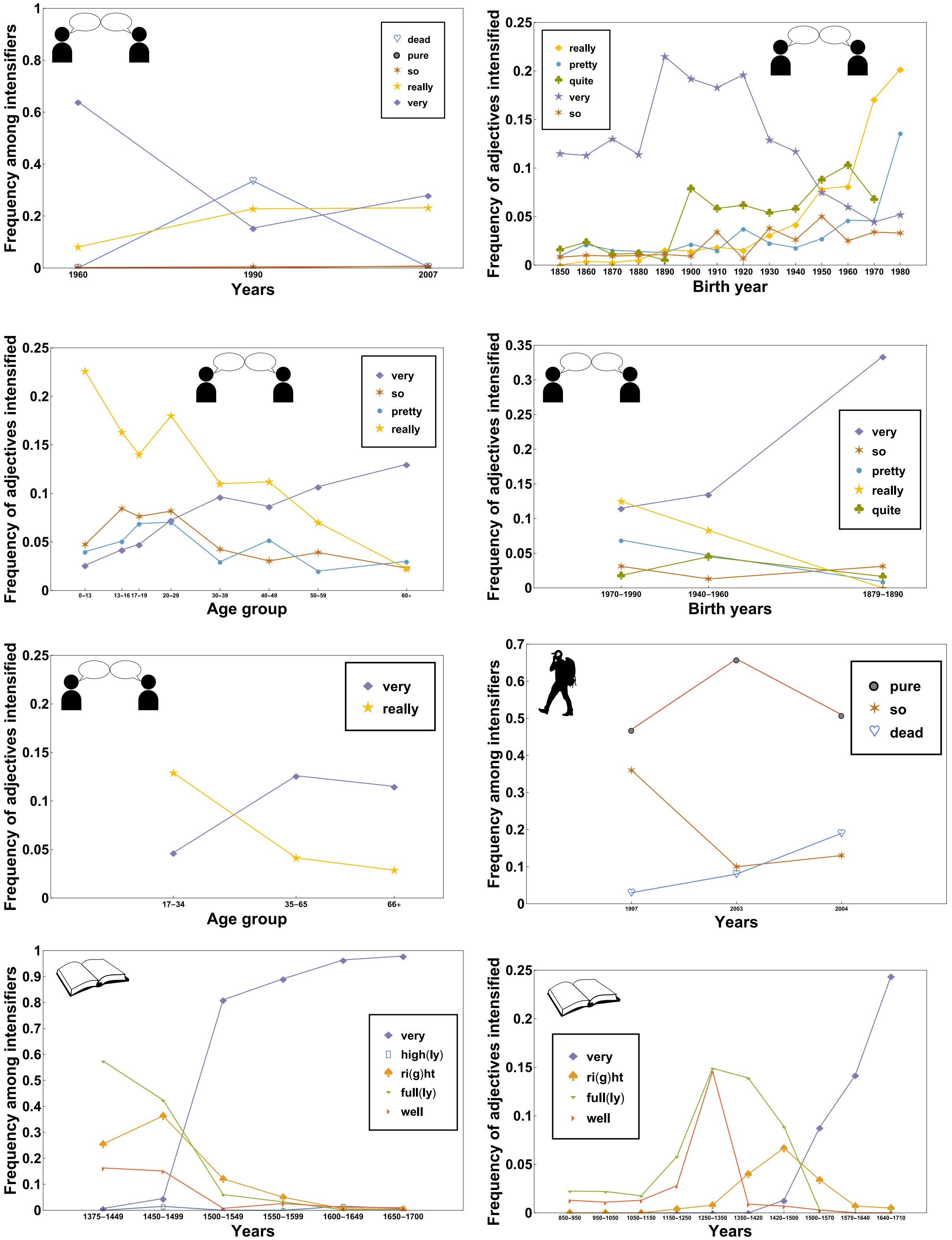

S1 Figure F. Instances of recycling from corpora of spoken and written language. In lexical order: speech from Tyneside [8], New Zealand [9], Toronto [10], Victoria [11], York [12], teenage speech from Glasgow [13], a corpus of historical medical texts in British English [14], the Penn Corpus of Historical English [15].

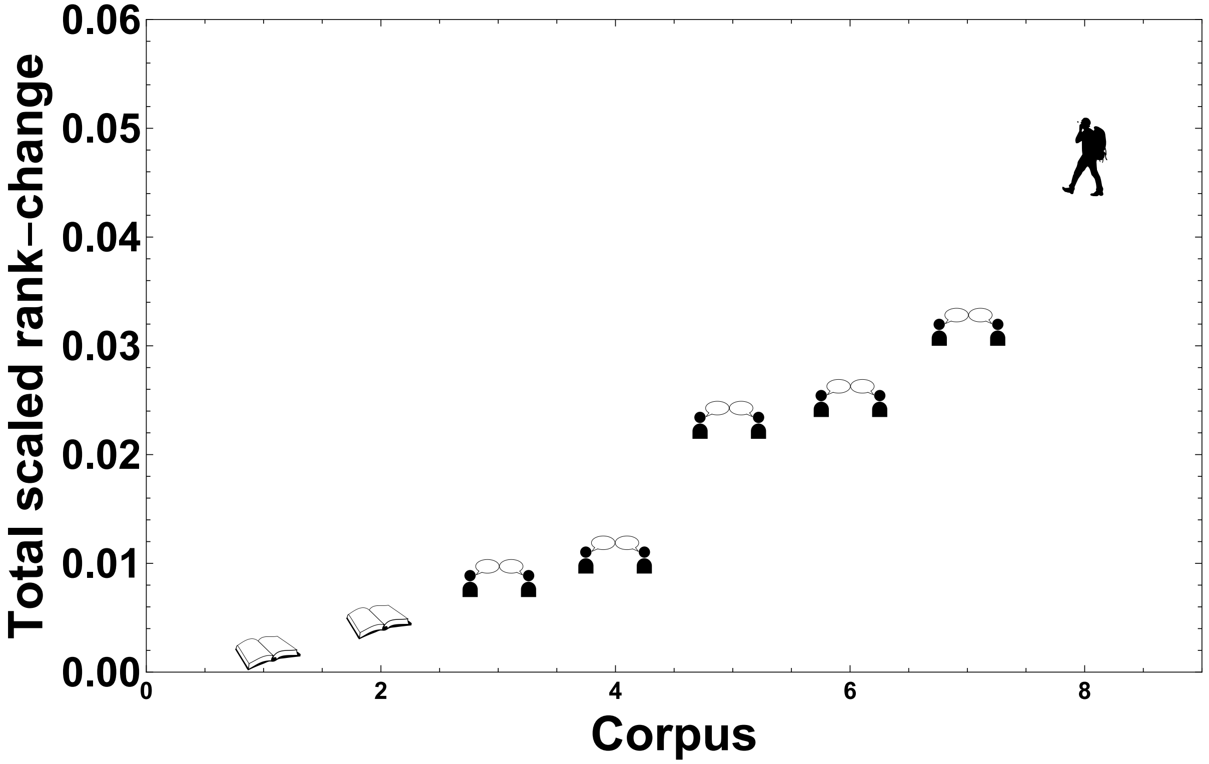

S1 Figure G. Measurements of rate of recycling in corpora. An approximation of the rate of recycling (per year) in real language. Each number is given by the number of rank-swaps in a corpus in Figure F (effectively, the number of line crossings in the figure), divided by the length of the study in years and the number of intensifiers measured in the study.

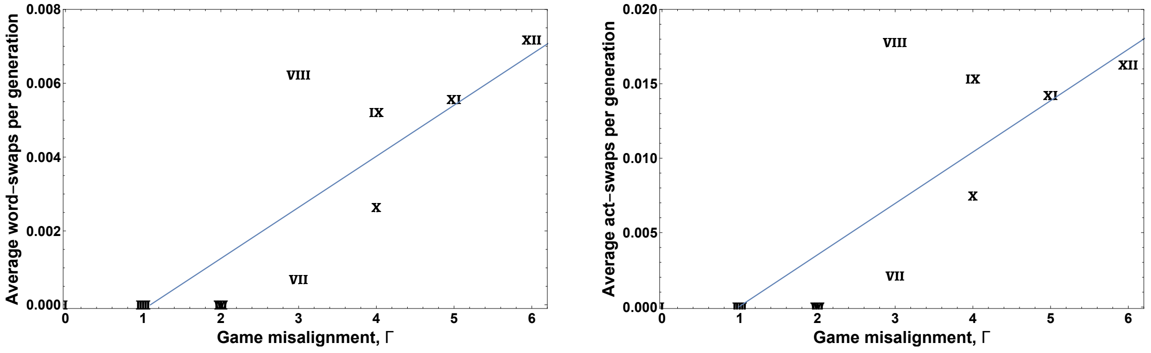

S1 Figure H. Meanings often change over time due to misaligned interests. (left): The equivalent of panel **(b)** in Fig 3 for the symmetric game. **(right):** The equivalent of panel **(d)** in Fig 3 for the symmetric game. The overlapping game symbols for $\Gamma = 1$ are **II,III**, and for $\Gamma = 2$ are **IV,V,VI**.

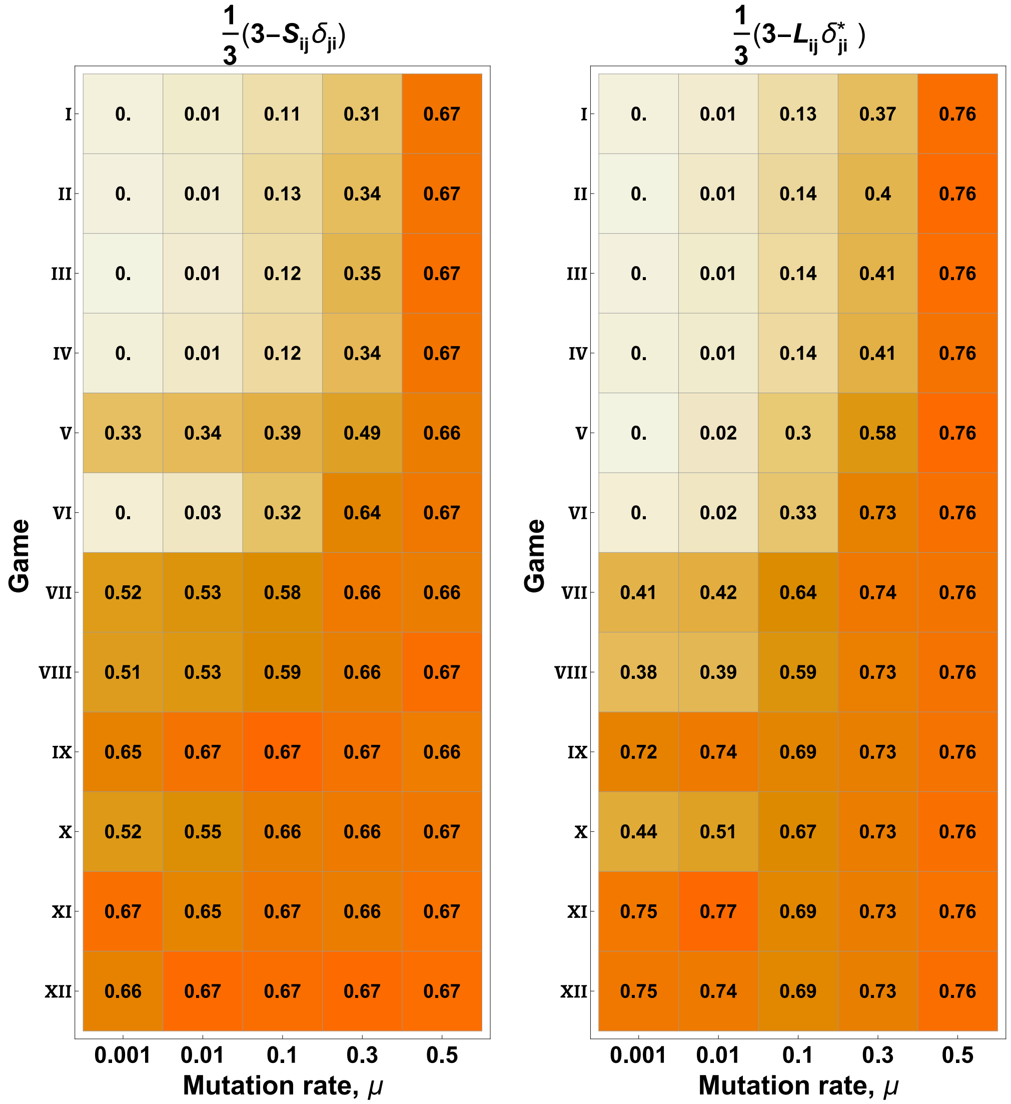

S1 Figure I. Original meaning of words is lost with increasing misalignment. The equivalent of Fig 4 for the symmetric game.

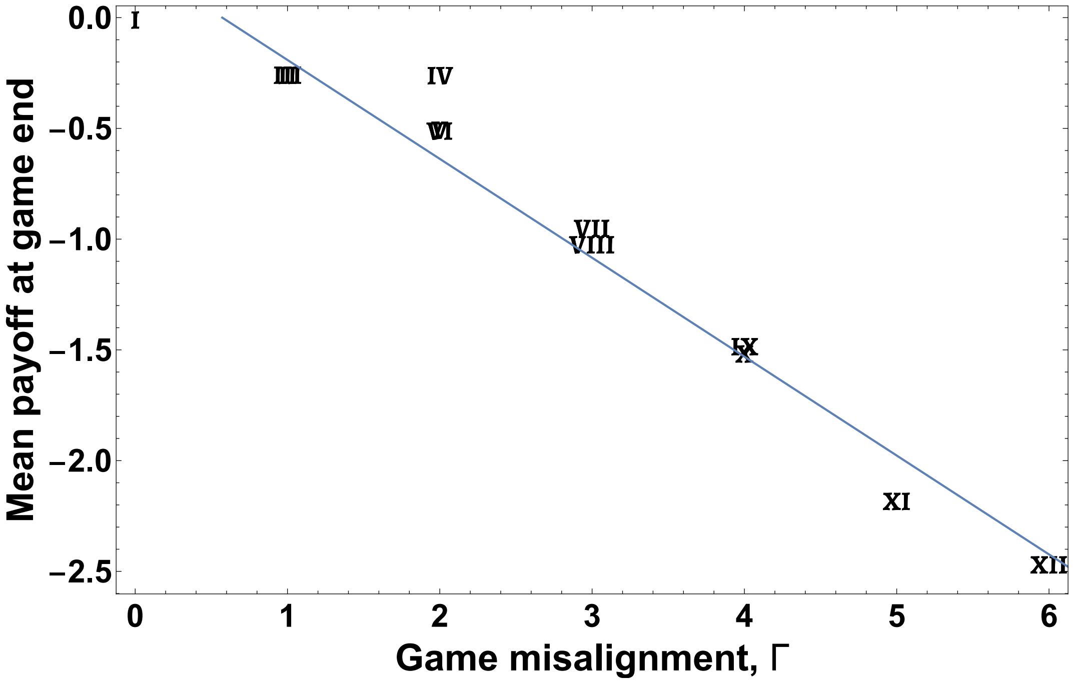

S1 Figure J. Misalignment of interests still leads to pessimal communication. Here we show the mean payoff of players at the end of 5,000 generations in the symmetric game with $k = 10$ and $\mu = 0.001$. Blue lines indicate linear best-fits. The overlapping game symbols for $\Gamma = 1$ are **II,III**, and for $\Gamma = 2$ are **V,VI**.

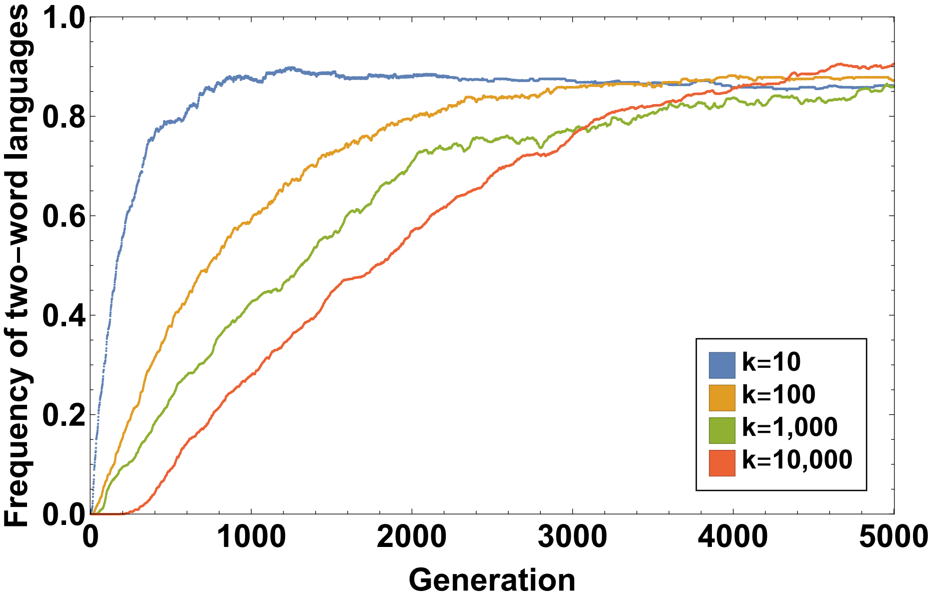

S1 Figure K. The role of the "learning parameter", k. In the main text we discuss and plot results for $k = 10$ only. This is because the role of k seems not to affect the long-term results (provided simulations are run for sufficiently many generations), and only control the "speed" of the population. As an example, we show here the invasion of informative languages by two-word languages in game **VIII** over time with $\mu = 0.001$ and all four values of k used.

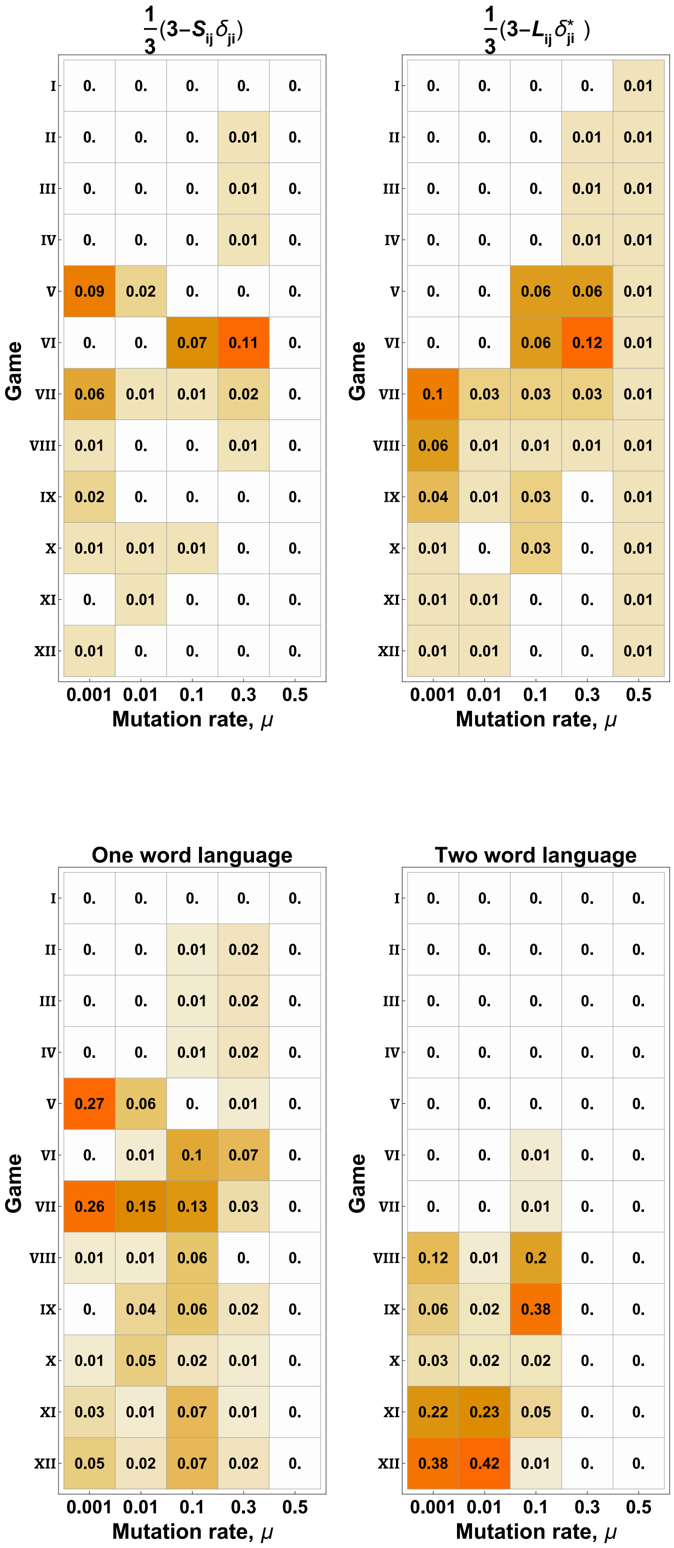

S1 Figure L. The "learning parameter" k has a negligible effect on most metrics discussed in the main text. Here, heatmaps indicate the maximum difference seen in end-of-game values of the total change in speaker and listener responses (top row) and the frequencies of one- and two-word languages (bottom row) for different values of $k \in [10, 100, 1000, 10000]$. In the few cases where these values exceed 0.1, they are always between $k = 10,000$ and $k = 1,000$, suggesting that they represent instances where $5,000$ generations is not enough for the slow-learning populations to converge to their long-term mutation-selection balances.

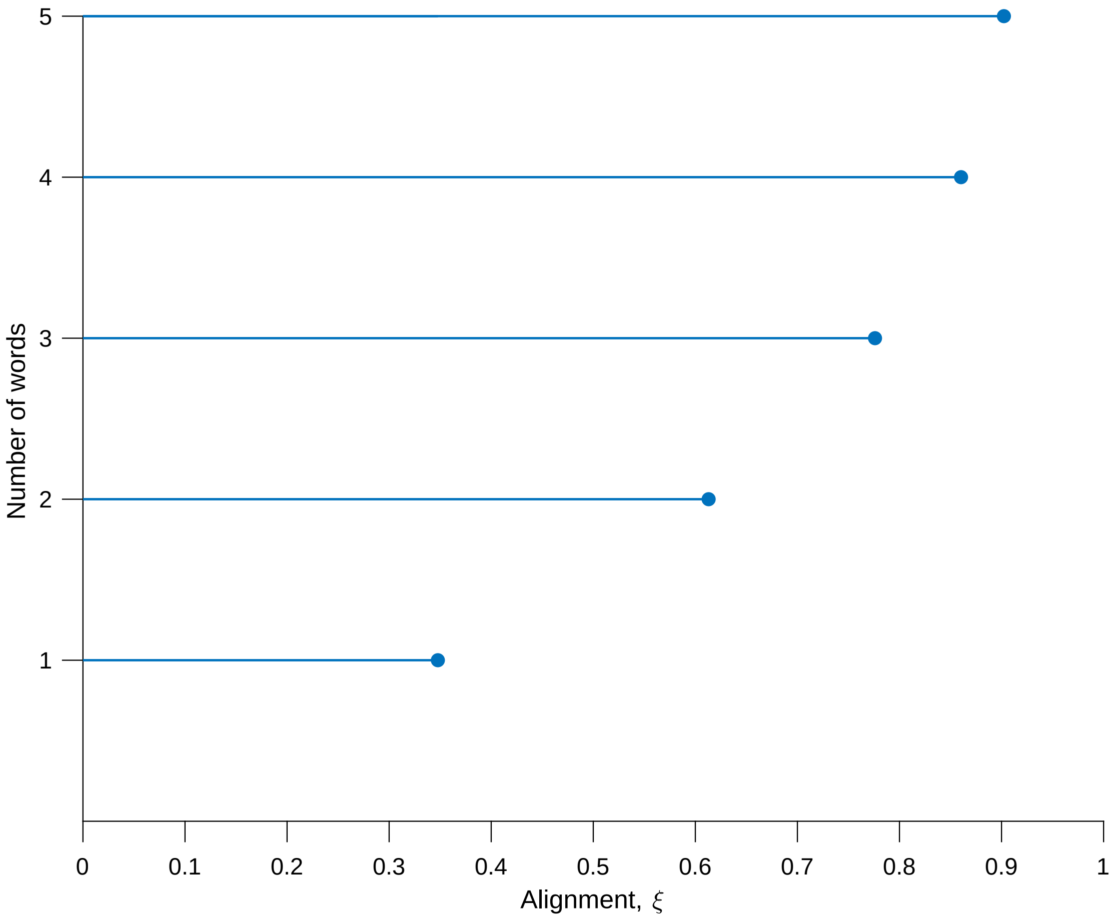

S1 Figure M. The maximum possible number of words in a (Bayesian) Nash equilibrium for models with multiplicative misalignment. This figure represents the equivalent of S1 Fig A for the alternative class of models contained in the **Supplementary Information**, for multiplicative misalignment where the speaker's optimal action is $\bar{a}_S = \gamma s = s/\xi$. As in the additive case, the maximum lexicon size drops off very quickly from infinity to five words, and the vast majority of cases are treated by a lexicon that has at most three words.

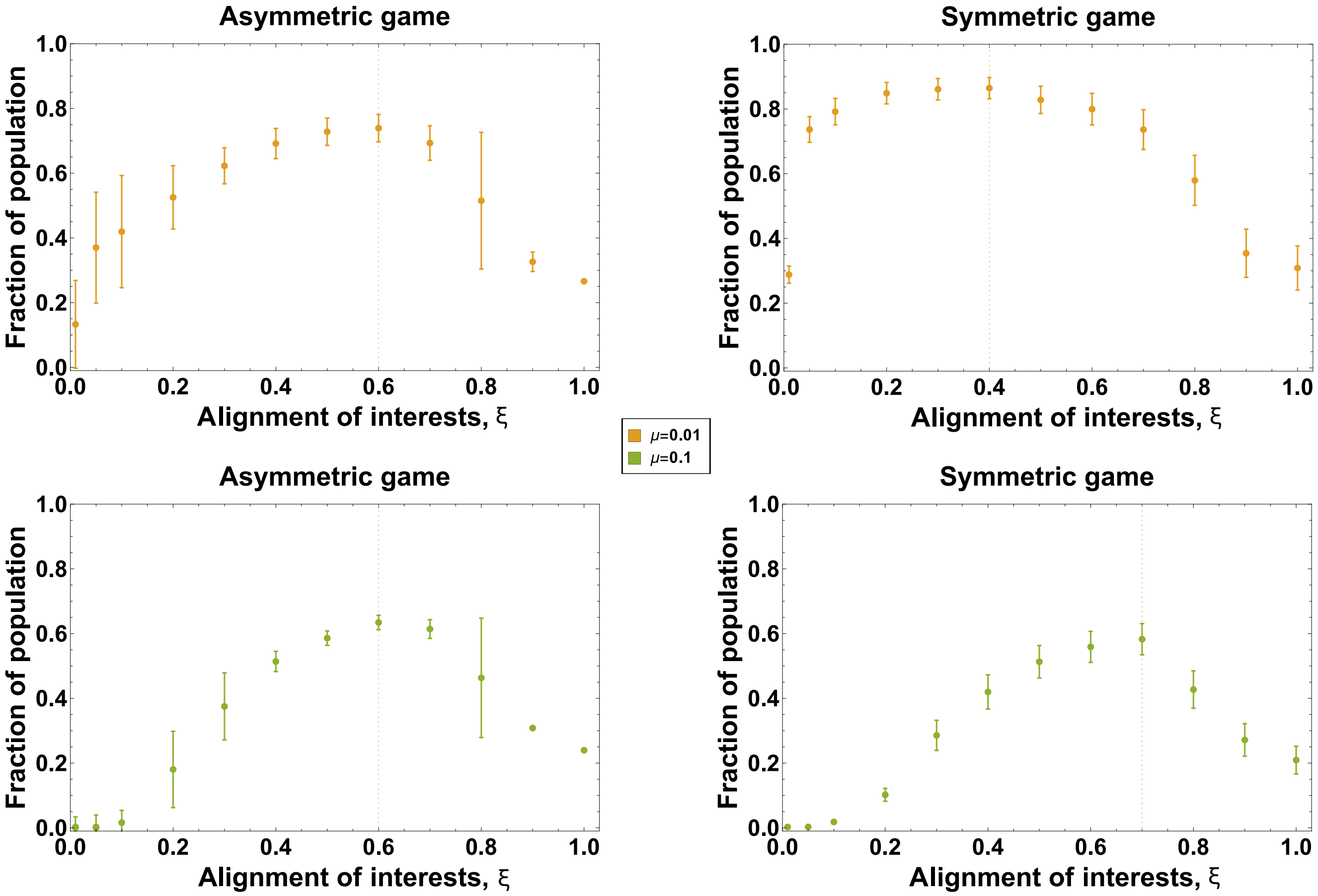

S1 Figure N. The transition between three-, two-, and one-word languages in the alternative model with multiplicative misalignment. Similar to the behavior of the additive class of models (Fig 3 and S1 Fig D), the multiplicative models show a steady transition in both asymmetric and symmetric modes of play between lexica with 3, 2, and finally 1-word languages as the alignment of interests between the two players decreases. Here, dots give the mean fraction of the population with a 2-word language at the end of realizations, and bars give the standard deviation. In all but the symmetric game with $\mu = 0.01$, the maximum fraction of 2-word lexica is achieved very close to $\xi = 0.61$, which is exactly where the Nash equilibrium lexicon size jumps from three words to two; on either side of this border, the rest of the population speaks either a three-word ($\xi > 0.61$) or one-word ($\xi < 0.61$) language.

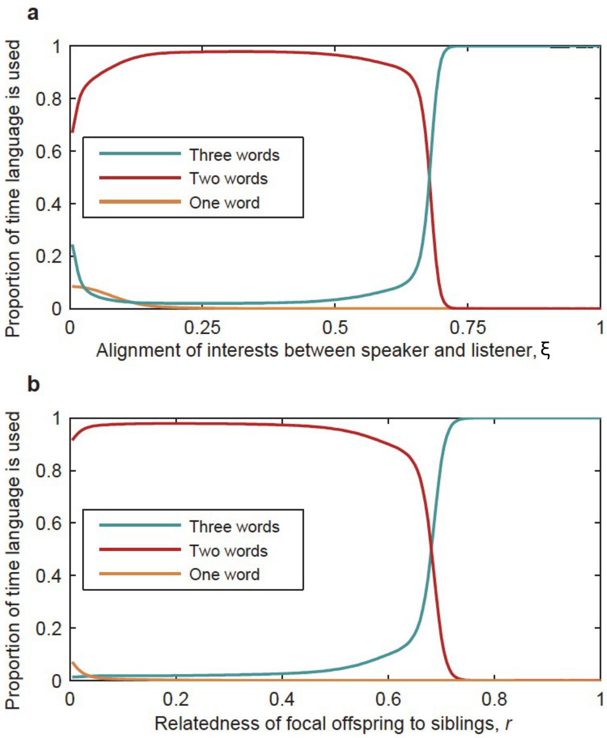

S1 Figure O. Analytical solution of the asymmetric game for the alternative model with multiplicative misalignment, as well as the associated Godfray chick-signalling game. Here, we show how our model(s) and their treatment can extend directly to other signaling games. Using tools previously derived to analytically solve pure-strategy asymmetric games in finite-size populations [7], we solved both our multiplicative asymmetric game for a finite-sized population of 100 players with pure strategies **(a)**, but also **(b)** the Godfray signalling game (see **Supplementary Information**) [3] for nutritional begging among offspring with different degrees of relatedness. The change in game, as well as the similarity between relatedness r in the Godfray game and the alignment ξ in our games, suggest that these results may generalize across signaling scenarios with misalignment of interest.

References

1. Crawford VP, Sobel J. Strategic information transmission. Econometrica. 1982;50:1431.

2. Crawford VP, Sobel J. Strategic Information Transmission. Econometrica. 1982 2021/05/12/;50(6):1431-51.

3. Godfray HCJ. Signalling of need by offspring to their parents. Nature. 1991;352:328.

4. Maynard Smith J. Honest signalling: the Philip Sidney game. Animal Behavior. 1991;42:1034.

5. Trivers RL. Parent-offspring conflict. American Zoologist. 1974;14:249.

6. Wright S. Coefficients of Inbreeding and Relationship. The American Naturalist. 1922;56(645):330-8. Available from: `https://doi.org/10.1086/279872`.

7. Veller C, Hayward LK. Finite-population evolution with rare mutations in asymmetric games. Journal of Economic Theory. 2016;162:93-113.

8. Barnfield K, Buchstaller I. In: Intensifiers on Tyneside. vol. 31; 2010. p. 252-87.

9. D'Arcy A. Stability, stasis and change: The longue durée of intensification. Diachronica. 2015;32(4):449-93.

10. Tagliamonte S. So different and pretty cool! Recycling intensifiers in Toronto, Canada. English Language and Linguistics. 2008;12(2):361–394.

11. Merx IM. Very jolly and really wild: Development in Victoria English intensifiers; 2018. .

12. Ito R, Tagliamonte S. Well weird, right dodgy, very strange, really cool: Layering and recycling in English intensifiers. Language in Society. 2003;32(2):257–279.

13. Macaulay R. Pure grammaticalization: The development of a teenage intensifier. Language Variation and Change. 2006;18(3):267–283.

14. Méndez-Naya B, Pahta P. Intensifiers in competition: The picture from early English medical writing; 2010. .

15. Méndez-Naya B. Co-occurrence and iteration of intensifiers in Early English. English Text Construction. 2017;10(2):249-73.
